# Synthesis, Antimicrobial, and Anti-inflammatory Activities of Novel 5-(1-Adamantyl)-4-arylideneamino-3-mercapto-1,2,4-triazoles and Related Derivatives

**DOI:** 10.3390/molecules15042526

**Published:** 2010-04-09

**Authors:** Mohamed A. Al-Omar, Ebtehal S. Al-Abdullah, Ihsan A. Shehata, Elsayed E. Habib, Tarek M. Ibrahim, Ali A. El-Emam

**Affiliations:** 1 Department of Pharmaceutical Chemistry, College of Pharmacy, King Saud University, Riyadh 11451, Saudi Arabia; 2 Department of Microbiology, Faculty of Pharmacy, University of Mansoura, Mansoura 35516, Egypt; 3 Department of Pharmacology and Toxicology, Faculty of Pharmacy, University of Mansoura, Mansoura 35516, Egypt

**Keywords:** 1-adamantyl derivatives, 1,2,4-triazoles, antimicrobial activity, anti-inflammatory activity

## Abstract

The reaction of 5-(1-adamantyl)-4-amino-3-mercapto-1,2,4-triazole (**5**) with various aromatic aldehydes in ethanol or acetic acid yielded the corresponding 4-arylideneamino derivatives **6a–v**. Treatment of the 4-(2,6-difluoro- and dichlorobenzylideneamino) derivatives **6o** and **6q** with 1-substituted piperazines, and formaldehyde solution in ethanol afforded good yields of the corresponding 5-(1-adamantyl)-4-(2,6-dihalobenzylideneamino-2-(4-substituted-1-piperazinylmethyl)-1,2,4-triazoline-3-thiones **7a–p**. 5-(1-Adamantyl)-4-arylideneamino-2-(4-ethoxycarbonyl-1-piperidylmethyl)-1,2,4-triazoline-3-thiones **8a–n**, were similarly prepared *via* the reaction of the corresponding arylideneamino derivative with ethyl 4-piperidinecarboxylate and formaldehyde solution in ethanol. Compounds **6a–v**, **7a–p** and **8a–n** were tested for *in vitro* activities against a panel of Gram-positive and Gram-negative bacteria and the yeast-like pathogenic fungus *Candida albicans*. Several derivatives showed good or moderate activities, particularly against the tested Gram-positive bacteria. In addition, the *in vivo* anti-inflammatory activity of 21 compounds was determined using the carrageenan-induced paw oedema method in rats. Compounds **7d**, **7g**, **7i, 7j**, **7l**, **8c**, **8e** and **8l** showed good or moderate dose-dependent activity in this area.

## 1. Introduction

Nonsteroidal anti-inflammatory drugs (NSAIDs) are among the most widely used therapeutics, primarily for the treatment of pain and inflammation in arthritis. The development of microbial resistance has necessitated the search for new potent antibacterial and antifungal agents. Derivatives of adamantane which have long been known for their antiviral activity against the influenza [1,2,3] and HIV viruses [4,5,6,7], were also associated with central nervous [8,9,10], antimicrobial [11,12,13,14,15] and anti-inflammatory activities [14,15,16,17,18]. 1,2,4-Triazole derivatives were reported to possess diverse biological activities, such as antibacterial [19,20,21] and anti-inflammatory [22,23,24,25] properties. In addition, several 5-substituted-4-arylideneamino-3-mercapto-1,2,4-triazoles and their piperazinomethyl derivatives were reported to exhibit significant antimicrobial activity [26,27,28]. In continuation to our interest in the chemical and pharmacological properties of adamantane derivatives [6,11,14,15,16], we report herein the synthesis, antimicrobial and anti-inflammatory activities of a new series of 5-(1-adamantyl)-4-arylideneamino-3-mercapto-1,2,4-triazoles and their piperazinomethyl and piperidinomethyl Mannich bases.

## 2. Results and Discussion

### 2.1. Chemistry

**Scheme 1:**
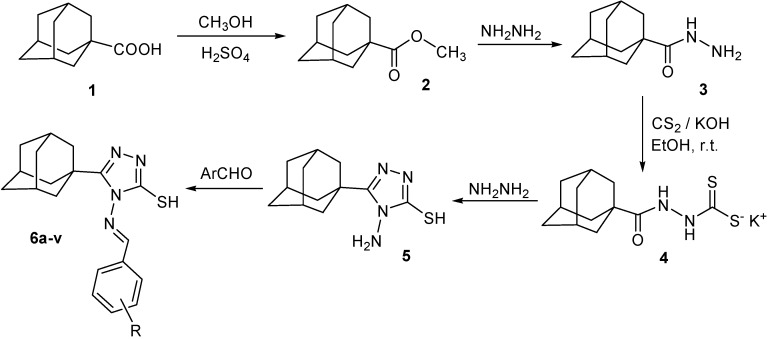
Synthetic Pathway for Compounds **6a–v**.

5-(1-Adamantyl)-4-amino-3-mercapto-1,2,4-triazole (**5**) was prepared starting from adamantane-1-carboxylic acid (**1**) *via* esterification with methanol to yield the methyl ester **2**, which was subsequently reacted with hydrazine to yield adamantane-1-carboxylic acid hydrazide (**3**). Treatment of **3** with carbon disulphide in ethanolic potassium hydroxide yielded the corresponding potassium *N*'-(1-adamantylcarbonyl)dithiocarbazate (**4**), which was cyclized by heating with hydrazine to yield 5-(1-adamantyl)-4-amino-3-mercapto-1,2,4-triazole (**5**) in good overall yield [29]. Treatment of compound **5** with the appropriate aromatic aldehydes under reflux in ethanol for 5 h yielded the corresponding arylideneamino derivatives **6a-v** in reasonable yields. In the cases of the reactions with 2-nitro-, 4-nitro-, 2,4-dichloro-, 3,4-dichloro-, 2,4-dinitro-, or 4,5-dimethoxy-2-nitrobenzaldehyde, the yields were very poor. This may be attributed to the poor solubility of these aldehydes in ethanol. However, carrying out the reaction in acetic acid, in which these aldehydes are freely soluble, in addition to its higher boiling point, greatly increased the yield (Scheme 1, Table 1).

**Scheme 2:**
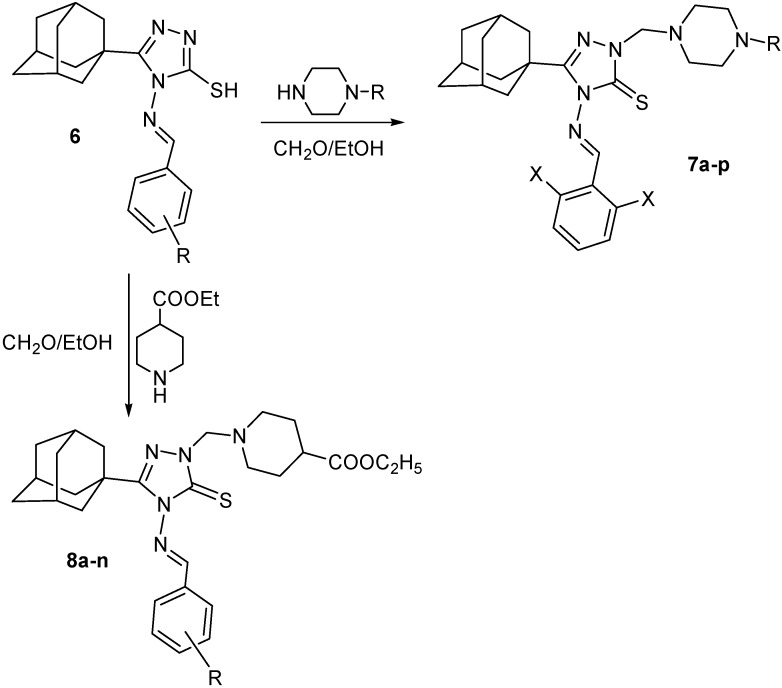
Synthetic Pathway for Compounds **7a–p** and **8a–n**.

The 2,6-difluoro- and dichlorobenzylideneamino derivatives **6o** and **6q** were reacted with several 1-substituted piperazines and formaldehyde solution in ethanol to yield the corresponding *N*-Mannich bases **7a–p** in good yields. The reaction was carried out by heating the reactants in ethanol for 15 min to enhance the solubility of compounds **6o** and **6q**. On monitoring the reaction with thin layer chromatography (TLC), the starting compounds **6o** and **6q** disappeared completely after 15 minutes and the products either precipitated on standing or after addition of water. The 5-(1-adamantyl)-4-arylideneamino-2-(4-ethoxycarbonyl-1-piperidylmethyl)-1,2,4-triazoline-3-thione *N*-Mannich bases **8a–n** were similarly prepared *via* the reaction of the corresponding 5-(1-adamantyl)-4-arylideneamino-3-mercapto-1,2,4-triazole with ethyl 4-piperidinecarboxylate and formaldehyde solution by heating the reactants in ethanol for 20 min (Scheme 2, Table 2 and Table 3). The structures of newly synthesized compounds **6a–v**, **7a–p** and **8a–n** were confirmed by ^1^H-NMR, ^13^C-NMR and mass spectra.

**Table 1:** Melting points, crystallization solvents, yields, molecular formulae and molecular weights of compounds **6a–v**.

Comp. No.	R	Mp (ºC)	Cryst. Solv.	Yield (%)	Molecular Formula (Mol. Wt.)
**6a**	H	222-224	EtOH/H_2_O	81	C_19_H_22_N_4_S (338.47)
**6b**	2-F	226-228	EtOH	74	C_19_H_21_FN_4_S (356.46)
**6c**	4-F	255-257	EtOH	71	C_19_H_21_FN_4_S (356.46)
**6d**	2-Cl	223-225	EtOH	75	C_19_H_21_ClN_4_S (372.91)
**6e**	4-Cl	207-209	AcOH	68	C_19_H_21_ClN_4_S (372.91)
**6f**	4-Br	228-230	EtOH/H_2_O	59	C_19_H_21_BrN_4_S (417.37)
**6g**	2-OH	238-240	EtOH	79	C_19_H_22_N_4_OS (354.47)
**6h**	4-OH	264-266	EtOH	82	C_19_H_22_N_4_OS (354.47)
**6i**	4-CH_3_	208-210	EtOH/H_2_O	80	C_20_H_24_N_4_S (352.50)
**6j**	2-OCH_3_	226-228	EtOH	77	C_20_H_24_N_4_OS (368.50)
**6k**	4-OCH_3_	214-216	EtOH	81	C_20_H_24_N_4_OS (368.50)
6l*	2-NO_2_	226-228	EtOH	52	C_19_H_21_N_5_O_2_S (383.47)
6m*	4-NO_2_	242-244	AcOH	50	C_19_H_21_N_5_O_2_S (383.47)
**6n**	4-(CH_3_)_2_N	223-225	EtOH	66	C_21_H_27_N_5_S (381.54)
**6o**	2,6-F_2_	241-243	EtOH	68	C_19_H_20_F_2_N_4_S (374.45)
**6p**	2-Cl,6-F	219-221	EtOH	62	C_19_H_20_ClFN_4_S (390.91)
**6q**	2,6-Cl_2_	207-209	DMF	49	C_19_H_20_Cl_2_N_4_S (407.36)
6r*	2,4-Cl_2_	233-235	AcOH	62	C_19_H_20_Cl_2_N_4_S (407.36)
6s*	3,4-Cl_2_	237-239	AcOH	69	C_19_H_20_Cl_2_N_4_S (407.36)
**6t**	3,4-(CH_3_O)_2_	191-193	EtOH	86	C_21_H_26_N_4_O_2_S (398.52)
6u*	2,4-(NO_2_)_2_	245-247	AcOH	49	C_19_H_20_N_6_O_4_S (428.46)
6v*	2-NO_2_,4,5-(CH_3_O)_2_	145-147	AcOH	68	C_21_H_25_N_5_O_4_S (443.52)

* Prepared by method B.

**Table 2:** Melting points, crystallization solvents, yields, molecular formulae and molecular weights of compounds **7a–p**.

Comp. No.	X	R	Mp (°C)	Cryst. Solv.	Yield (%)	Molecular Formula (Mol. Wt.)
**7a**	F	CH_3_	102-104	EtOH/H_2_O	54	C_25_H_32_F_2_N_6_S (486.62)
**7b**	F	C_2_H_5_	169-171	EtOH/H_2_O	89	C_26_H_34_F_2_N_6_S (500.65)
**7c**	F	COOC_2_H_5_	156-158	EtOH/H_2_O	82	C_27_H_34_F_2_N_6_O_2_S (544.66)
**7d**	F	C_6_H_5_	151-153	EtOH	79	C_30_H_34_F_2_N_6_S (548.69)
**7e**	F	4-FC_6_H_4_	140-142	EtOH/H_2_O	80	C_30_H_33_F_3_N_6_S (566.68)
**7f**	F	3-CF_3_C_6_H_4_	132-134	EtOH/H_2_O	88	C_31_H_33_F_5_N_6_S (616.69)
**7g**	F	2-CH_3_OC_6_H_4_	184-186	EtOH/CHCl_3_	85	C_31_H_36_F_2_N_6_OS (578.72)
**7h**	F	C_6_H_5_CH_2_	148-150	EtOH	80	C_31_H_36_F_2_N_6_S (562.72)
**7i**	Cl	CH_3_	109-111	EtOH/H_2_O	68	C_25_H_32_Cl_2_N_6_S (519.53)
**7j**	Cl	C_2_H_5_	117-119	EtOH/H_2_O	75	C_26_H_34_Cl_2_N_6_S (533.56)
**7k**	Cl	COOC_2_H_5_	136-138	EtOH/H_2_O	72	C_27_H_34_Cl_2_N_6_O_2_S (577.57)
**7l**	Cl	C_6_H_5_	170-172	EtOH	83	C_30_H_34_Cl_2_N_6_S (581.60)
**7m**	Cl	4-FC_6_H_4_	184-186	EtOH	85	C_30_H_33_Cl_2_FN_6_S (599.59)
**7n**	Cl	3-CF_3_C_6_H_4_	159-161	EtOH	86	C_31_H_33_Cl_2_F_3_N_6_S (649.60)
**7o**	Cl	2-CH_3_OC_6_H_4_	139-141	EtOH	67	C_31_H_36_Cl_2_N_6_OS (611.63)
**7p**	Cl	C_6_H_5_CH_2_	178-180	EtOH	80	C_31_H_36_Cl_2_N_6_S (595.63)

**Table 3:** Melting points, crystallization solvents, yield percentages, molecular formulae and molecular weights of compounds **8a–n**.

Comp. No.	R	Mp (°C)	Cryst. Solv.	Yield (%)	Molecular Formula (Mol. Wt.)
**8a**	H	125-127	EtOH/H_2_O	82	C_28_H_37_N_5_O_2_S (507.69)
**8b**	2-F	134-136	EtOH/H_2_O	72	C_28_H_36_FN_5_O_2_S (525.68)
**8c**	2-Cl	158-160	EtOH	75	C_28_H_36_ClN_5_O_2_S (542.14)
**8d**	4-CH_3_	135-137	EtOH/H_2_O	92	C_29_H_39_N_5_O_2_S (521.72)
**8e**	2-OH	127-129	EtOH/H_2_O	79	C_28_H_37_N_5_O_3_S (523.69)
**8f**	4-OH	195-197	EtOH/H_2_O	84	C_28_H_37_N_5_O_3_S (523.69)
**8g**	4-OCH_3_	124-126	EtOH/H_2_O	88	C_29_H_39_N_5_O_3_S (537.72)
**8h**	2,6-F_2_	148-150	EtOH/H_2_O	69	C_28_H_35_F_2_N_5_O_2_S (543.67)
**8i**	2-Cl,6-F	148-150	EtOH	74	C_28_H_35_ClFN_5_O_2_S (560.13)
**8j**	2,6-Cl_2_	151-153	EtOH	75	C_28_H_35_Cl_2_N_5_O_2_S (576.58)
**8k**	2,4-Cl_2_	129-131	EtOH	68	C_28_H_35_Cl_2_N_5_O_2_S (576.58)
**8l**	3,4-Cl_2_	186-188	EtOH	71	C_28_H_35_Cl_2_N_5_O_2_S (576.58)
**8m**	3,4-(CH_3_O)_2_	113-115	EtOH/H_2_O	76	C_30_H_41_N_5_O_4_S (567.74)
**8n**	2-NO_2_,4,5-(CH_3_O)_2_	182-184	EtOH/CHCl_3_	80	C_30_H_40_N_6_O_6_S (612.74)

### 2.2. Antimicrobial Testing

The newly synthesized compounds **6a–v**, **7a–p** and **8a–n** were tested for their *in vitro* growth inhibitory activity against the standard strains of the Institute of Fermentation of Osaka (IFO) namely; *Staphylococcus aureus* IFO 3060, *Bacillus subtilis* IFO 3007, *Micrococcus luteus* IFO 3232 (Gram-positive bacteria), *Escherichia coli* IFO 3301, *Pseudomonas aeuroginosa* IFO 3448 (Gram-negative bacteria), and the yeast-like pathogenic fungus *Candida albicans* IFO 0583. The primary screening was carried out using the agar disc-diffusion method using Müller-Hinton agar medium [30]. The results of the preliminary antimicrobial testing of compounds **6a–v**, **7a–p** and **8a–n** (200 μg/disc), the antibacterial antibiotic ampicillin trihydrate (100 μg/disc) and the antifungal drug clotrimazole (100 μg/disc) are shown in Table 4. The antimicrobial activity results of the 4-arylideneaminotriazoles **6a–v** revealed that the aryl substituents greatly influenced the antimicrobial activity. The halo and hydroxyl derivatives were highly active particularly against the tested Gram-positive bacteria, while the nitro and methoxy derivatives were generally inactive. In addition, the hydroxy derivatives **6g** and **6h** showed marked activity against the tested Gram-negative bacteria. The 4-fluoro **6c** and 4-bromo **6f** derivatives were significantly active against *Candida albicans*. Based on the antibacterial activity of the arylideneamino derivatives **6a-v**, and the previously reported high chemotherapeutic activity of several 2,6-dihalophenyl derivatives [31,32,33], the 2,6-difluoro- and dichlorobenzylideneamino derivatives **6o** and **6q** were selected to prepare their 4-substituted-1-piperazinyl Mannich bases **7a–p**. The results of the antimicrobial activity of the 4-substituted-1-piperazinyl Mannich bases **7a–p** were generally lower than those of the arylideneamino derivatives **6a–v** but the specificity was almost similar. On the other hand, the antimicrobial activity of the 4-ethoxycarbonyl-1-piperidyl Mannich bases **8a–n** was higher than the 4-substituted-1-piperazinyl Mannich bases **7a–p**. The most potent member of these derivatives were the 4-hydroxybenzylidene **8f** and the 3,4-dimethoxybenzylidene **8m** derivatives which displayed strong broad spectrum activity. The minimal inhibitory concentration (MIC) for the most active compounds **6h**, **6o**, **7n**, **8a**, **8e**, **8f** and **8m** against the same microorganism used in the primary screening was determined using the microdilution susceptibility method in Müller-Hinton Broth and Sabouraud Liquid Medium [34]. The MIC of the most active compounds, the antibacterial antibiotic ampicillin trihydrate and the antifungal drug clotrimazole (Table 5) were in accordance with the results obtained in the primary screening.

**Table 4:** Antimicrobial activity of compounds **6a–v, 7a–p** and **8a–n** (200 μg/8 mm disc), the broad spectrum antibacterial drugs gentamicin (100 μg/8 mm disc), ampicillin (100 μg/8 mm disc) and the antifungal drug clotrimazole (100 μg/8 mm disc) against *Staphylococcus aureus* IFO 3060 (*SA*), *Bacillus subtilis* IFO 3007 (*BS*), *Micrococcus luteus* IFO 3232 (*ML*), *Escherichia coli* IFO 3301 (*EC*), *Pseudomonas aeuroginosa* IFO 3448 (*PA*), and *Candida albicans* IFO 0583 (*CA*).

Comp. No.	Diameter of Growth Inhibition Zone (mm)*					
	*SA*	*BS*	*ML*	*EC*	*PA*	*C A*
**6a**	10	13	-	-	-	-
**6b**	14	14	-	-	-	-
**6c**	16	17	-	-	-	17
**6d**	11	14	-	-	-	-
**6e**	14	16	-	-	-	-
**6f**	13	14	14	-	-	16
**6g**	14	16	12	15	13	-
**6h**	19	25	13	19	14	-
**6i**	15	18	15	-	-	-
**6j**	-	-	-	-	-	-
**6k**	-	-	-	-	-	-
**6l**	14	14	-	-	-	-
**6m**	-	-	-	-	-	-
**6n**	-	-	-	-	-	-
**6o**	18	19	-	-	-	-
**6p**	-	-	-	-	-	-
**6q**	14	16	-	-	-	-
**6r**	16	17	-	-	-	-
**6s**	17	17	-	-	-	12
**6t**	-	-	-	-	-	-
**6u**	-	-	-	-	-	-
**6v**	-	-	-	-	-	-
**7a**	13	15	-	-	-	-
**7b**	15	14	-	15	-	-
**7c**	11	15		-	-	-
**7d**	-	-	14	-	-	-
**7e**	-	-	-	-	12	-
**7f**	12	11	-	18	11	-
**7g**	-	-	-	-	-	-
**7h**	-	-	-	-	-	17
**7i**	12	11	-	-	-	-
**7j**	14	12	-	-	-	-
**7k**	-	11	-	-	-	-
**7l**	-	-	-	-	-	-
**7m**	13	12	-	-	-	-
**7n**	-	-	-	19	14	-
**7o**	-	-	-	-	-	-
**7p**	-	-	-	-	-	-
**8a**	12	19	16	-	-	-
**8b**	-	-	-	-	-	-
**8c**	-	-	-	15	-	-
**8d**	11	15	-	-	-	-
**8e**	17	22	-	-	-	13
**8f**	22	26	-	-	-	19
**8g**	15	11	-	-	-	-
**8h**	-	-	-	-	-	14
**8i**	14	15	-	-	-	-
**8j**	12	16	16	-	-	-
**8k**	15	17	-	-	-	-
**8l**	11	13	-	-	-	-
**8m**	20	24	17	19	16	-
**8n**	-	-	-	-	-	-
**Gentamicin**	26	25	18	20	19	NT
**Ampicillin**	23	21	19	17	16	NT
**Clotrimazole**	NT	NT	NT	NT	NT	21

* (-): Inactive (inhibition zone <10 mm). (NT): Not tested.

**Table 5:** The minimal inhibitory concentrations (MIC, μg/mL) of compounds **6h**, **6o**, **7n**, **8a**, **8e**, **8f**, **8m**, the broad spectrum antibacterial drugs gentamicin, ampicillin and the antifungal drug clotrimazole against *Staphylococcus aureus* IFO 3060 (*SA*), *Bacillus subtilis* IFO 3007 (*BS*), *Micrococcus luteus* IFO 3232 (*ML*), *Escherichia coli* IFO 3301(*EC*), *Pseudomonas aeuroginosa* IFO 3448 (*PA*), and *Candida albicans* IFO 0583 (*CA*).

Comp. No.	Minimal Inhibitory Concentration (MIC, g/mL)*					
	*SA*	*BS*	*ML*	*EC*	*PA*	*C A*
**6h**	2	1	ND	2	ND	ND
**6o**	ND	4	ND	ND	ND	ND
**7n**	ND	ND	ND	2	ND	ND
**8a**	ND	4	ND	ND	ND	ND
**8e**	ND	2	ND	ND	ND	ND
**8f**	2	1	ND	ND	ND	8
**8m**	2	1	ND	2	ND	ND
**Gentamicin**	2	2	2	0.5	1	ND
**Ampicillin**	2	0.5	2	2	2	ND
**Clotrimazole**	ND	ND	ND	ND	ND	2

* ND: Not determined.

### 2.3. Acute anti-inflammatory activity testing

The acute *in vivo* anti-inflammatory activity of 21 representative compounds (**6b**, **6e**, **6h**, **6j**, **6r**, **6t**, **7b**, **7d**, **7g**, **7j**, **7l**, **7m**, **7p**, **8a**, **8c**, **8e**, **8f**, **8h**, **8k**, **8l** and **8n**) was determined following the carrageenan-induced paw oedema method in rats [35]. The selection of the representative compounds and dose levels were made after carrying out pilot experiments which showed the absence of anti-inflammatory activity for some compounds and that the dose levels 20 and 40 mg/kg showed no signs of acute toxicity. The results of the anti-inflammatory activity of the tested compounds (20 & 40 mg/kg) and the potent anti-inflammatory drug Indomethacin (5 mg/kg) are listed in Table 6. The majority of the tested compounds showed varying degrees of activity. The highest activity was shown by compound **7l**, which produced strong dose-dependent inhibition of carrageenan-induced paw oedema (>50%), while compounds **7d** and **8c** were moderately active (30–50%) at 20 & 40 mg/kg dose level. Compounds **7g**, **7j**, **8e**, and **8l** were moderately active at 40 mg/kg dose level and weakly active at 20 mg/kg level. The structure-anti-inflammatory activity of the tested derivatives revealed that the triazole *N*-2 and *N*-4 substituents greatly influence the anti-inflammatory activity. 

**Table 6:** Anti-inflammatory effect of intraperitoneal injection of (20 & 40 mg/kg) of compounds **6b**, **6e**, **6h**, **6j**, **6r**, **6t**, **7b**, **7d**, **7g**, **7j**, **7l**, **7m**, **7p**, **8a**, **8c**, **8e**, **8f**, **8h**, **8k**, **8l**, **8n** and Indomethacin (5 mg/kg) against carrageenan-induced paw oedema in rats.

Comp. No.	Mean % Reduction of paw oedema from control^a^	
	20 mg/kg	40 mg/kg
Control^b^	0 (± 0.04)	
**6b**	-5.50 (± 0.13)^*^	-2.71 (± 0.12)^*^
**6e**	3.16 (± 0.11)^*^	-8.56 (± 0.07)^*^
**6h**	13.28 (± 0.15)^**^	11.29 (± 0.14)^**^
**6j**	11.06 (± 0.13)^**^	13.91 (± 0.11)^**^
**6r**	10.32 (± 0.09)^**^	10.54 (± 0.11)^**^
**6t**	21.92 (± 0.08)^**^	19.37 (± 0.07)^**^
**7b**	17.88 (± 0.11)^**^	20.01 (± 0.09)^**^
**7d**	39.16 (± 0.09)^**^	39.88 (± 0.89)^**^
**7g**	25.88 (± 0.07)^**^	38.37 (± 0.07)^**^
**7j**	27.95 (± 0.12)^**^	34.76 (± 0.10)^**^
**7l**	28.86 (± 0.08)^**^	50.44 (± 0.06)^**^
**7m**	13.06 (± 0.08)^**^	22.90 (± 0.08)^**^
**7p**	2.05 (± 0.06)^*^	4.99 (± 0.08)^*^
**8a**	23.23 (± 0.02)^**^	15.27 (± 0.05)^**^
**8c**	33.02 (± 0.11)^**^	37.66 (± 0.14)^**^
**8e**	20.41 (± 0.07)^**^	31.20 (± 0.09)^**^
**8f**	-18.25 (± 0.13)	1.36 (± 0.04)
**8h**	-20.21 (± 0.11)	-9.80 (± 0.07)
**8k**	9.91 (± 0.133)^*^	6.81 (± 0.09)^*^
**8l**	10.45 (± 0.10)^**^	35.01 (± 0.12)^**^
**8n**	-13.75 (± 0.18)^*^	-11.61 (± 0.10)^*^
**Indomethacin (5 mg/kg)**	52.79 (± 0.04)	

^a^ Results are expressed as mean % inhibition ± S.E.M. (n = 5) and compared with student “*t*” test. ^b^ The group was injected with 1 ml of 0.5% aqueous carboxymethyl cellulose solution. ^*^ Inactive: Significantly different from Indomethacin at p < 0.05. ^**^ Activity comparable to Indomethacin (significantly different from Indomethacin at p < 0.05.

The *N*-2 unsubstituted adamantyltriazoles **6b**, **6e**, **6h**, **6j**, **6r** and **6t** were weakly active or completely inactive, while the *N*-2 piperazinomethyl derivatives **7b**, **7d**, **7g**, **7j**, **7l**, **7m** and **7p** were generally active. The activity was also found to be dependent on the nature of the 4-arylideneamino and the 4-piperazinyl substituents. The activity of the 2,6-dichlorobenzylidene derivatives were slightly higher than their 2,6-difluoro-benzylidene analogues. It could be also concluded that the phenyl substituents are better compared with the ethyl, 4-fluorophenyl, 2-methoxyphenyl and benzyl substituents. The replacement of the 4-substituted-1-piperazinyl moiety with a 4-carbethoxy-1-piperidyl moiety resulted in marked decrease in activity, only the chloro derivatives **8c** and **8l** and the 2-hydroxy derivative **8e** exhibited moderate activity. There are in fact a high number of enzyme/receptors involved in the inflammatory process. Without specific tests it is quite difficult to hypothesize the mechanism of action of the tested compounds, they may exert their action *via* inhibition of the cyclooxygenase enzymes like other nonsteroidal anti-inflammatory agents. In addition, the recently reported activity of some adamantane derivatives as selective inhibitors of 11β-hydroxysteroid dehydrogenase type 1 (11β-HSD1) [36,37] should be taken inconsideration. The 11β-hydroxysteroid dehydrogenase type 1 converts cortisone to the active glucocorticoid cortisol, which is responsible for various metabolic disorders including water retention, thus the inhibition of 11β-HSD1 would result in increasing the intracellular cortisone level.

## 3. Experimental

### 3.1. General

Melting points (ºC) were measured in open glass capillaries using a Branstead 9001 Electrothermal melting point apparatus and are uncorrected. NMR spectra were obtained on a Bruker AC 500 Ultra Shield NMR spectrometer (Fällanden, Switzerland) operating at 500.13 MHz for ^1^H and 125.76 MHz for ^13^C; the chemical shifts are expressed in δ (ppm) downfield from tetramethylsilane (TMS) as internal standard; coupling constants (*J*) are expressed in Hz. Electrospray ionization mass spectra (ESI-MS) were recorded on a Waters QuatroMicro triple quadrupole tandem mass spectrometer at 4.0 and 3.5 kV for positive and negative ions, respectively. Elemental analyses (C, H, N, S) were in full agreement with the proposed structures within ±0.4% of the theoretical values. Monitoring the reactions and checking the purity of the final products were carried out by thin layer chromatography (TLC) using silica gel precoated aluminum sheets (60 F_254_, Merck) and visualization with ultraviolet light (UV) at 365 and 254 nm. The bacterial strains and *Candida albicans* fungus were obtained from the Institute of Fermentation of Osaka (IFO), Osaka, Japan. The reference drugs ampicillin trihydrate (CAS 7177-48-2), clotrimazole (CAS 23593-75-1) and indomethacin (CAS 53-86-1) were obtained from Sigma-Aldrich Chemie GmbH, Taufkirchen, Germany. The Sprauge-Dawley rats were purchased from local animal house (Abu-Rawash, Giza, Egypt). The animal experiments for the determination of the anti-inflammatory activity were carried out in agreement with the pertinent legal and ethical standards of the international guidelines.

### 3.2. 5-(1-Adamantyl)-4-arylideneamino-3-mercapto-1,2,4-triazoles ***6a–v***

*Method A:* A mixture of the appropriate aromatic aldehyde (2.0 mmol) and 5-(1-adamantyl)-4-amino-3-mercapto-1,2,4-triazole (**5**, 0.5 g, 2.0 mmol) in absolute ethanol (10 mL) was heated under reflux for 5 h. On cooling, the separated solid was filtered, washed with cold ethanol (5 mL), dried and crystallized (Table 1). *Method B*(compounds **6l**, **6m**, **6r**, **6s**, **6u** and **6v**): A mixture of the appropriate aromatic aldehyde (2.0 mmol) and 5-(1-adamantyl)-4-amino-3-mercapto-1,2,4-triazole (**5**, 0.5 g, 2 mmol) in acetic acid (8 mL) was heated under reflux for 4 h. On cooling, the separated solid was filtered, washed with cold ethanol (5 mL), dried and crystallized (Table 1).

*5-(1-Adamantyl)-4-benzylideneamino-3-mercapto-1,2,4-triazole*** 6a**: ^1^H-NMR (DMSO-d_6_): δ 1.69–1.73 (m, 6H, adamantane-H), 2.02 (s, 3H, adamantane-H), 2.07 (s, 6H, adamantane-H), 7.58–7.65 (m, 3H, Ar-H), 7.92 (d, 2H, Ar-H, *J* = 7.0 Hz), 9.71 (s, 1H, CH=N), 13.82 (s, 1H, SH). ^13^C-NMR: 27.72, 35.28, 36.47, 38.61 (adamantane-C), 128.94, 129.78, 132.67, 133.17 (Ar-C), 156.04, 162.50 (triazole C-5 & CH=N), 165.02 (triazole C-3). MS, *m/z* (Rel. Int.): 338 (M^+^, 51), 261 (12), 236 (26), 235 (100), 221 (14), 202 (17), 135 (15), 104 (12), 90 (11), 77 (6).

*5-(1-Adamantyl)-4-(2-fluorobenzylideneamino)-3-mercapto-1,2,4-triazole* **6b**: 1H-NMR (DMSO-d_6_): δ 1.71 (s, 6H, adamantane-H), 2.02 (s, 3H, adamantane-H), 2.08 (s, 6H, adamantane-H), 7.40–7.45 (m, 2H, Ar-H), 7.67–7.71 (m, 1H, Ar-H), 8.04 (t, 1H, Ar-H, *J* = 7.0 Hz), 10.18 (s, 1H, CH=N), 13.85 (s, 1H, SH). ^13^C-NMR: 27.77, 35.36, 36.49, 38.37 (adamantane-C), 117.0, 120.45, 125.92, 127.86, 135.25, 156.88 (Ar-C), 156.20, 162.44 (triazole C-5 & CH=N), 163.18 (triazole C-3). MS, *m/z* (Rel. Int.): 356 (M^+^, 44), 339 (25), 297 (17), 266(24), 260 (20), 235 (100), 234 (60), 220 (31), 135 (25), 122 (26), 108 (29), 96 (10).

*5-(1-Adamantyl)-4-(4-fluorobenzylideneamino)-3-mercapto-1,2,4-triazole*** 6c**: ^1^H-NMR (DMSO-d_6_): δ 1.71 (s, 6H, adamantane-H), 2.01 (s, 3H, adamantane-H), 2.07 (s, 6H, adamantane-H), 7.34 (d, 2H, Ar-H, *J* = 6.5 Hz), 7.94 (d, 2H, Ar-H, *J* = 6.5 Hz), 9.71 (s, 1H, CH=N), 13.81 (s, 1H, SH). ^13^C-NMR: 27.73, 35.28, 36.47, 38.63 (adamantane-C), 116.40, 129.28, 131.09, 163.95 (Ar-C), 156.02, 162.54 (triazole C-5 & CH=N), 165.37 (triazole C-3). MS, *m/z* (Rel. Int.): 356 (M^+^, 17), 235 (100), 234 (58), 220 (14), 135 (34), 122 (22), 121 (62), 107 (18), 95 (44).

*5-(1-Adamantyl)-4-(2-chlorobenzylideneamino)-3-mercapto-1,2,4-triazole* **6d**: 1H-NMR (DMSO-d_6_): δ 1.73 (s, 6H, adamantane-H), 2.03 (s, 3H, adamantane-H), 2.09 (s, 6H, adamantane-H), 7.58–7.70 (m, 3H, Ar-H), 8.15 (d, 1H, Ar-H, *J* = 7.5 Hz), 10.48 (s, 1H, CH=N), 13.89 (s, 1H, SH). ^13^C-NMR: 27.76, 35.41, 36.51, 38.70 (adamantane-C), 127.77, 128.64, 130.40, 130.96, 134.46, 135.69 (Ar-C), 156.27, 158.73 (triazole C-5 & CH=N), 162.44 (triazole C-3). MS, *m/z* (Rel. Int.): 374 (M^+^ +2, 25), 372 (M^+^, 100), 337 (34), 261 (25), 249 (26), 235 (58), 234 (48), 221 (20), 140 (32), 135 (21), 125 (26), 110 (12).

*5-(1-Adamantyl)-4-(4-chlorobenzylideneamino)-3-mercapto-1,2,4-triazole*** 6e**: ^1^H-NMR (DMSO-d_6_): δ 1.70 (s, 6H, adamantane-H), 2.01 (s, 3H, adamantane-H), 2.06 (s, 6H, adamantane-H), 7.66 (d, 2H, Ar-H, *J* = 6.5 Hz), 7.93 (d, 2H, Ar-H, *J* = 6.5 Hz), 9.76 (s, 1H, CH=N), 13.81 (s, 1H, SH). ^13^C-NMR: 27.73, 35.29, 36.46, 38.63 (adamantane-C), 129.98, 130.56, 131.56, 137.89 (Ar-C), 156.04, 162.53 (triazole C-5 & CH=N), 163.66 (triazole C-3). MS, *m/z* (Rel. Int.): 374 (M^+^ +2, 10), 373 (M^+^ +1, 40), 372 (M^+^, 23), 371 (100).

*5-(1-Adamantyl)-4-(2-bromobenzylideneamino)-3-mercapto-1,2,4-triazole*** 6f**: ^1^H-NMR (DMSO-d_6_): δ 1.71 (s, 6H, adamantane-H), 2.01 (s, 3H, adamantane-H), 2.09 (s, 6H, adamantane-H), 7.99 (d, 2H, Ar-H, *J* = 8.5), 7.86 (d, 2H, Ar-H, *J* = 8.5 Hz), 9.75 (s, 1H, CH=N), 13.82 (s, 1H, SH). ^13^C-NMR: 27.80, 35.30, 36.53, 38.64 (adamantane-C), 126.89, 130.71, 131.89, 132.93 (Ar-C), 156.04, 162.51 (triazole C-5 & CH=N), 162.73 (triazole C-3). MS, *m/z* (Rel. Int.): 418 (M^+^ +2, 20), 417 (M^+^ +1, 100), 416 (M^+^, 20), 415 (100).

*5-(1-Adamantyl)-4-(2-hydroxybenzylideneamino)-3-mercapto-1,2,4-triazole*** 6g**: ^1^H-NMR (DMSO-d_6_): δ 1.69–1.73 (m, 6H, adamantane-H), 2.01 (s, 3H, adamantane-H), 2.07 (s, 6H, adamantane-H), 6.97–7.02 (m, 2H, Ar-H), 7.42–7.46 (m, 1H, Ar-H), 7.90–7.92 (m, 1H, Ar-H), 9.91 (s, 1H, CH=N), 10.55 (s, 1H, OH), 13.46 (s, 1H, SH). ^13^C-NMR: 27.74, 35.29, 36.47, 38.61 (adamantane-C), 117.26, 118.95, 120.26, 127.24, 134.72, 158.97 (Ar-C), 156.01, 161.63 (triazole C-5 & CH=N), 162.49 (triazole C-3). MS, *m/z* (Rel. Int.): 355 (M^+^ +1, 7), 354 (M^+^, 28), 353 (100).

*5-(1-Adamantyl)-4-(4-hydroxybenzylideneamino)-3-mercapto-1,2,4-triazole*** 6h**: ^1^H-NMR (DMSO-d_6_): δ 1.67–1.71 (m, 6H, adamantane-H), 2.01 (s, 3H, adamantane-H), 2.06 (s, 6H, adamantane-H), 6.94 (d, 2H, Ar-H, *J* = 8.5 Hz), 7.76 (d, 2H, Ar-H, *J* = 8.5 Hz), 9.36 (s, 1H, CH=N), 10.38 (s, 1H, OH), 13.70 (s, 1H, SH). ^13^C-NMR: 27.73, 35.21, 36.48, 38.57 (adamantane-C), 116.65, 123.51, 131.18, 162.51 (Ar-C), 155.91, 162.32 (triazole C-5 & CH=N), 165.79 (triazole C-3). MS, *m/z* (Rel. Int.): 355 (M^+^ +1, 6), 354 (M^+^, 26), 353 (100).

*5-(1-Adamantyl)-4-(4-methylbenzylideneamino)-3-mercapto-1,2,4-triazole*** 6i**: ^1^H-NMR (DMSO-d_6_): δ 1.69–1.73 (m, 6H, adamantane-H), 2.01 (s, 3H, adamantane-H), 2.06 (s, 6H, adamantane-H), 2.40 (s, 3H, CH_3_), 7.39 (d, 2H, Ar-H, *J* = 7.5 Hz), 7.81 (d, 2H, Ar-H, *J* = 7.5 Hz), 9.61 (s, 1H, CH=N), 13.77 (s, 1H, SH). ^13^C-NMR: 21.72 (CH_3_), 27.73, 35.26, 36.47, 38.60 (adamantane-C), 128.96, 129.99, 130.37, 143.57 (Ar-C), 156.0, 162.51 (triazole C-5 & CH=N), 165.19 (triazole C-3). MS, *m/z* (Rel. Int.): 351 (M^+^ -1, 14), 234 (100), 233 (32).

*5-(1-Adamantyl)-4-(2-methoxybenzylideneamino)-3-mercapto-1,2,4-triazole*** 6j**: ^1^H-NMR (DMSO-d_6_): δ 1.67–1.74 (m, 6H, adamantane-H), 2.02 (s, 3H, adamantane-H), 2.07 (s, 6H, adamantane-H), 3.89 (s, 3H, OCH_3_), 7.13–7.23 (m, 2H, Ar-H), 7.60–7.63 (m, 1H, Ar-H), 8.01 (d, 1H, Ar-H, *J* = 8.0 Hz), 10.02 (s, 1H, CH=N), 13.76 (s, 1H, SH). ^13^C-NMR: 27.74, 35.30, 36.49, 38.60 (adamantane-C), 56.54 (OCH_3_), 112.99, 120.74, 121.59, 126.69, 134.97, 159.84 (Ar-C), 156.13 (triazole C-5), 159.81 (CH=N), 162.39 (triazole C-3). MS, *m/z* (Rel. Int.): 369 (M^+^ +1, 7), 368 (M^+^, 23), 367 (100).

*5-(1-Adamantyl)-4-(4-methoxybenzylideneamino)-3-mercapto-1,2,4-triazole*** 6k**: ^1^H-NMR (DMSO-d_6_): δ 1.67–1.73 (m, 6H, adamantane-H), 2.01 (s, 3H, adamantane-H), 2.06 (s, 6H, adamantane-H), 3.86 (s, 3H, OCH_3_), 7.13 (d, 2H, Ar-H, *J* = 8.5 Hz), 7.87 (d, 2H, Ar-H, *J* = 8.5 Hz), 9.50 (s, 1H, CH=N), 13.79 (s, 1H, SH). ^13^C-NMR: 27.73, 35.24, 36.48, 38.59 (adamantane-C), 56.04 (OCH_3_), 115.32, 125.11, 130.91, 163.43 (Ar-C), 155.95, 162.50 (triazole C-5 & CH=N), 165.12 (triazole C-3). MS, *m/z* (Rel. Int.): 369 (M^+^ +1, 29), 368 (M^+^, 76), 353 (33), 259 (33), 249 (37), 236 (51), 235 (49), 234 (29), 135 (54), 133 (100), 108 (112).

*5-(1-Adamantyl)-4-(2-nitrobenzylideneamino)-3-mercapto-1,2,4-triazole*** 6l**: ^1^H-NMR (DMSO-d_6_): δ 1.72 (s, 6H, adamantane-H), 2.01 (s, 3H, adamantane-H), 2.07 (s, 6H, adamantane-H), 7.85 (t, 1H, Ar-H, *J* = 7.5 Hz), 7.94 (t, 1H, Ar-H, *J* = 7.5 Hz), 8.17 (d, 2H, Ar-H, *J* = 8.0 Hz), 10.43 (s, 1H, CH=N), 13.90 (s, 1H, SH). ^13^C NMR: 27.76, 35.39, 36.42, 38.63 (adamantane-C), 125.40, 126.87, 129.43, 133.54, 134.53, 149.29 (Ar-C), 156.26, 159.20 (triazole C-5 & CH=N), 162.65 (triazole C-3). MS, *m/z* (Rel. Int.): 384 (M^+^ +1, 7), 383 (M^+^, 24), 382 (100).

*5-(1-Adamantyl)-4-(4-nitrobenzylideneamino)-3-mercapto-1,2,4-triazole*** 6m**: ^1^H-NMR (DMSO-d_6_): δ 1.71 (s, 6H, adamantane-H), 2.01 (s, 3H, adamantane-H), 2.06 (s, 6H, adamantane-H), 7.89 (d, 2H, Ar-H, *J* = 8.4 Hz), 8.32(d, 2H, Ar-H, *J* = 8.4 Hz), 10.21 (s, 1H, CH=N), 13.88 (s, 1H, SH). ^13^C NMR: 27.73, 35.21, 36.40, 38.61 (adamantane-C), 124.12, 128.83, 136.55, 150.22 (Ar-C), 155.66, 161.26 (triazole C-5 & CH=N), 162.85 (triazole C-3).

*5-(1-Adamantyl)-4-(4-dimethylaminobenzylideneamino)-3-mercapto-1,2,4-triazole*** 6n**: ^1^H-NMR (DMSO-d_6_): δ 1.66–1.71 (m, 6H, adamantane-H), 2.01 (s, 3H, adamantane-H), 2.06 (s, 6H, adamantane-H), 3.04 (s, 6H, CH_3_), 6.83 (d, 2H, Ar-H, *J* = 8.5 Hz), 7.71 (d, 2H, Ar-H, *J* = 9.0 Hz), 9.21 (s, 1H, CH=N), 13.74 (s, 1H, SH). ^13^C NMR: 27.74, 35.18, 36.50, 38.55 (adamantane-C), 39.89 (CH_3_), 112.20, 119.31, 130.66, 153.72 (Ar-C), 155.88, 162.51 (triazole C-5 & CH=N), 165.99 (triazole C-3). MS, *m/z* (Rel. Int.): 382 (M^+^ +1, 8), 381 (M^+^, 36), 379 (100).

*5-(1-Adamantyl)-4-(2,6-difluorobenzylideneamino)-3-mercapto-1,2,4-triazole*** 6o**: ^1^H-NMR (DMSO-d_6_): δ 1.71 (s, 6H, adamantane-H), 2.01 (s, 3H, adamantane-H), 2.09 (s, 6H, adamantane-H), 7.30–7.34 (m, 2H, Ar-H), 7.67-7.72 (m, 1H, Ar-H), 10.37 (s, 1H, CH=N), 13.87 (s, 1H, SH). ^13^C NMR: 27.30, 34.89, 35.87, 37.67 (adamantane-C), 109.74, 112.67, 134.86, 160.06 (Ar-C), 155.87, 160.06 (triazole C-5 & CH=N), 162.11 (triazole C-3). MS, *m/z* (Rel. Int.): 375 (M^+^ +1, 10), 374 (M^+^, 33), 343 (14), 236 (37), 235 (100), 234 (43), 220 (17), 139 (40), 135 (18), 126 (18), 113 (13).

*5-(1-Adamantyl)-4-(2-chloro-6-fluorobenzylideneamino)-3-mercapto-1,2,4-triazole*** 6p**: ^1^H-NMR (DMSO-d_6_): δ 1.71 (s, 6H, adamantane-H), 2.01 (s, 3H, adamantane-H), 2.09 (s, 6H, adamantane-H), 7.44–7.48 (m, 1H, Ar-H), 7.52 (d, 1H, Ar-H, *J* = 8.0 Hz), 7.63–7.67 (m, 1H, Ar-H), 10.54 (s, 1H, CH=N), 13.89 (s, 1H, SH). ^13^C NMR: 27.79, 35.41, 36.36, 38.23 (adamantane-C), 116.52, 119.14, 127.17, 134.57, 135.99, 162.59 (Ar-C), 156.41, 160.51 (triazole C-5 & CH=N), 162.21 (triazole C-3). MS, *m/z* (Rel. Int.): 392 (M^+^ +2, 8), 390 (M^+^, 20), 371 (12), 355 (22), 336 (9), 236 (56), 235 (100), 234 (18), 220 (26), 156 (33), 135 (14), 129 (7), 127 (22).

*5-(1-Adamantyl)-4-(2,6-dichlorobenzylideneamino)-3-mercapto-1,2,4-triazole*** 6q**: ^1^H-NMR (DMSO-d_6_): δ 1.68 (s, 6H, adamantane-H), 1.99 (s, 3H, adamantane-H), 2.09 (s, 6H, adamantane-H), 7.53–7.68 (m, 3H, Ar-H), 10.63 (s, 1H, CH=N), 13.93 (s, 1H, SH). ^13^C NMR: 27.77, 35.48, 36.38, 38.38 (adamantane-C), 128.33, 130.42, 133.60, 135.32 (Ar-C), 156.43, 158.38 (triazole C-5 & CH=N), 162.46 (triazole C-3). MS, *m/z* (Rel. Int.): 410 (M^+^ +4, 4), 409 (M^+^ +3, 16), 408 (M^+^ +2, 18), 407 (M^+^ +1, 74), 406 (M^+^, 22), 405 (100).

*5-(1-Adamantyl)-4-(2,4-dichlorobenzylideneamino)-3-mercapto-1,2,4-triazole*** 6r**: ^1^H-NMR (DMSO-d_6_): δ 1.72 (s, 6H, adamantane-H), 2.03 (s, 3H, adamantane-H), 2.08 (s, 6H, adamantane-H), 7.67–7.69 (m, 1H, Ar-H), 7.84 (s, 1H, Ar-H), 8.12 (d, 1H, Ar-H, *J* = 8.0 Hz), 10.50 (s, 1H, CH=N), 13.89 (s, 1H, SH). ^13^C NMR: 27.76, 35.41, 36.49, 38.71 (adamantane-C), 128.91, 129.12, 129.49, 130.48, 136.44, 138.29 (Ar-C), 156.26, 157.41 (triazole C-5 & CH=N), 162.44 (triazole C-3). MS, *m/z* (Rel. Int.): 410 (M^+^ +4, 3), 409 (M^+^ +3, 15), 408 (M^+^ +2, 17), 407 (M^+^ +1, 69), 406 (M^+^, 22), 405 (100).

*5-(1-Adamantyl)-4-(3,4-dichlorobenzylideneamino)-3-mercapto-1,2,4-triazole*** 6s**: ^1^H-NMR (DMSO-d_6_): δ 1.70–1.72 (m, 6H, adamantane-H), 2.02 (s, 3H, adamantane-H), 2.06 (s, 6H, adamantane-H), 7.85–7.93 (m, 2H, Ar-H), 8.13 (s, 1H, Ar-H), 9.88 (s, 1H, CH=N), 13.86 (s, 1H, SH). ^13^C NMR: 27.74, 35.33, 36.47, 38.67 (adamantane-C), 128.34, 130.61, 132.19, 132.74, 133.40, 135.64 (Ar-C), 156.09, 161.65 (triazole C-5 & CH=N), 162.56 (triazole C-3). MS, *m/z* (Rel. Int.): 410 (M^+^ +4, 3), 409 (M^+^ +3, 15), 408 (M^+^ +2, 17), 407 (M^+^ +1, 73), 406 (M^+^, 23), 405 (100).

*5-(1-Adamantyl)-4-(3,4-dimethoxybenzylideneamino)-3-mercapto-1,2,4-triazole*** 6t**: ^1^H-NMR (DMSO-d_6_): δ 1.67–1.74 (m, 6H, adamantane-H), 2.02 (s, 3H, adamantane-H), 2.08 (s, 6H, adamantane-H), 3.84 (s, 3H, OCH_3_), 3.86 (s, 3H, OCH_3_), 7.15 (d, 1H, Ar-H, *J* = 8.5 Hz), 7.47 (d, 1H, Ar-H, *J* = 8.5 Hz), 7.50 (s, 1H, Ar-H), 9.56 (s, 1H, CH=N), 13.74 (s, 1H, SH). ^13^C NMR: 27.76, 35.28, 36.52, 38.64 (adamantane-C), 56.0 (OCH_3_), 56.28 (OCH_3_), 110.09, 112.34, 124.20, 125.25, 149.74, 153.35 (Ar-C), 156.01, 162.46 (triazole C-5 & CH=N), 164.54 (triazole C-3). MS, *m/z* (Rel. Int.): 399 (M^+^ +1, 7), 398 (M^+^, 24), 397 (100).

*5-(1-Adamantyl)-4-(2,4-dinitrobenzylideneamino)-3-mercapto-1,2,4-triazole*** 6u**: ^1^H-NMR (DMSO-d_6_): δ 1.72 (s, 6H, adamantane-H), 2.02 (s, 3H, adamantane-H), 2.08 (s, 6H, adamantane-H), 8.41 (d, 1H, Ar-H, *J* = 8.5 Hz), 8.65 (d, 1H, Ar-H, *J* = 8.5 Hz), 9.03 (s, 1H, Ar-H), 11.51 (s, 1H, CH=N), 13.46 (s, 1H, SH). ^13^C NMR: 27.80, 34.80, 36.52, 38.36 (adamantane-C), 120.79, 128.01, 129.52, 131.72, 135.05, 147.74 (Ar-C), 157.09, 157.86 (triazole C-5 & CH=N), 167.73 (triazole C-3). MS, *m/z* (Rel. Int.): 451 (M^+^ +Na, 4), 450 (11), 451 (48), 436 (100), 428 (M^+^, 3), 427 (10).

*5-(1-Adamantyl)-4-(4,5-dimethoxy-2-nitrobenzylideneamino)-3-mercapto-1,2,4-triazole*** 6v**: ^1^H-NMR (DMSO-d_6_): δ 1.70–1.74 (m, 6H, adamantane-H), 2.02 (s, 3H, adamantane-H), 2.10 (s, 6H, adamantane-H), 3.96 (s, 3H, OCH_3_), 3.97 (s, 3H, OCH_3_), 7.64 (s, 1H, Ar-H), 7.75 (s, 1H, Ar-H), 10.56 (s, 1H, CH=N), 13.86 (s, 1H, SH). ^13^C NMR: 27.77, 35.44, 36.49, 38.76 (adamantane-C), 56.67 (OCH_3_), 57.05 (OCH_3_), 108.73, 109.16, 120.95, 143.12, 151.89, 153.10 (Ar-C), 156.28, 157.92 (triazole C-5 & CH=N), 162.56 (triazole C-3). MS, *m/z* (Rel. Int.): 444 (M^+^ +1, 7), 443 (M^+^, 24), 442 (100).

### 5-(1-Adamantyl)-4-arylideneamino-2-(4-substituted-1-piperazinylmethyl)-1,2,4-triazoline-3-thiones ***7a–p***

A mixture of the 5-(1-adamantyl)-4-(2,6-dihalobenzylideneamino)-3-mercapto-1,2,4-triazoles **6o** or **6q** (1.0 mmol), the appropriate *N*-substituted piperazine (1.0 mmol) and 37% formaldehyde solution (1 mL), in ethanol (8 mL), was heated under reflux for 15 min when a clear solution was obtained. Stirring was continued for 12 h at room temperature and the mixture was allowed to stand overnight. Cold water (5 mL) was added and the reaction mixture was stirred for 20 min. The precipitated crude products were filtered, washed with water, dried, and crystallized (Table 2).

*5-(1-Adamantyl)-4-(2,6-difluorobenzylideneamino)-2-(4-methyl-1-piperazinylmethyl)-1,2,4-triazoline-3-thione* **7a**: ^1^H-NMR (CDCl_3_): 1.78 (s, 6H, adamantane-H), 2.08 (s, 3H, adamantane-H), 2.16 (s, 6H, adamantane-H), 2.30 (s, 3H, CH_3_), 2.47 (s, 4H, piperazine-H), 2.92 (s, 4H, piperazine-H), 5.18 (s, 2H, CH_2_), 7.02–7.07 (m, 2H, Ar-H), 7.46–7.48 (m. 1H, Ar-H), 10.64 (s, 1H, CH=N). ^13^C NMR: 27.95, 35.51, 36.45, 38.32 (adamantane-C), 46.05 (CH_3_), 55.05, 58.36 (piperazine-C), 68.68 (CH_2_), 110.87, 112.16, 133.21, 152.24 (Ar-C), 155.52, 160.99 (triazole C-5 & CH=N), 163.05 (C=S). MS, *m/z* (Rel. Int.): 488 (M^+^ +2, 30), 487 (M^+^ +1, 100).

*5-(1-Adamantyl)-4-(2,6-difluorobenzylideneamino)-2-(4-ethyl-1-piperazinylmethyl)-1,2,4-triazoline-3-thione* **7b**: ^1^H-NMR (CDCl_3_): δ 1.07 (t, 3H, CH_3_, *J* = 7.0 Hz), 1.78 (s, 6H, adamantane-H), 2.08 (s, 3H, adamantane-H), 2.16 (s, 6H, adamantane-H), 2.40 (q, 2H, C***H***_2_CH_3_, *J* = 7.0 Hz), 2.48–2.49 (m, 4H, piperazine-H), 2.92 (s, 4H, piperazine-H), 5.18 (s, 2H, CH_2_), 7.02 (t, 2H, Ar-H, *J* = 8.5 Hz), 7.44–7.48 (m. 1H, Ar-H), 10.62 (s, 1H, CH=N). ^13^C NMR: 11.91 (CH_3_), 28.0, 35.50, 36.46, 38.33 (adamantane-C), 52.32 (***C***H_2_CH_3_), 50.42, 52.80 (piperazine-C), 68.79 (CH_2_), 110.89, 112.16, 133.20, 152.22 (Ar-C), 155.45, 161.0 (triazole C-5 & CH=N), 163.14 (C=S). MS, *m/z* (Rel. Int.): 502 (M^+^ +2, 32), 501 (M^+^ +1, 100).

*5-(1-Adamantyl)-4-(2,6-difluorobenzylideneamino)-2-(4-ethoxycarbonyl-1-piperazinylmethyl)-1,2,4-triazoline-3-thione* **7c**: ^1^H-NMR (CDCl_3_): δ 1.24 (t, 3H, CH_3_, *J* = 7.0 Hz), 1.79 (s, 6H, adamantane-H), 2.09 (s, 3H, adamantane-H), 2.17 (s, 6H, adamantane-H), 2.81 (s, 4H, piperazine-H), 3.50 (s, 4H, piperazine-H), 4.10 (q, 2H, C***H***_2_CH_3_, *J* = 7.0 Hz), 5.16 (s, 2H, CH_2_), 7.02 (t, 2H, Ar-H, *J* = 7.0 Hz), 7.46–7.50 (m. 1H, Ar-H), 10.64 (s, 1H, CH=N). ^13^C NMR: 14.62 (CH_3_), 27.98, 35.55, 36.43, 38.35 (adamantane-C), 50.37, 52.39 (piperazine-C), 61.32 (***C***H_2_CH_3_), 68.97 (CH_2_), 110.81, 112.22, 133.29, 152.23 (Ar-C), 155.46, 155.70, 161.04 (C=O, triazole C-5 & CH=N), 163.20 (C=S). MS, *m/z* (Rel. Int.): 546 (M^+^ +2, 6), 445 (M^+^ +1, 20).

*5-(1-Adamantyl)-4-(2,6-difluorobenzylideneamino)-2-(4-phenyl-1-piperazinylmethyl)-1,2,4-triazoline-3-thione* **7d**: ^1^H-NMR (CDCl_3_): δ 1.80 (s, 6H, adamantane-H), 2.10 (s, 3H, adamantane-H), 2.19 (s, 6H, adamantane-H), 3.04 (s, 4H, piperazine-H), 3.23 (s, 4H, piperazine-H), 5.24 (s, 2H, CH_2_), 6.89 (t, 1H, Ar-H, *J* = 7.0 Hz), 6.94 (d, 2H, Ar-H, *J* = 8.0 Hz), 7.03 (t. 2H, Ar-H, *J* = 8.5 Hz), 7.26–7.28 (m, 2H, Ar-H), 7.47–7.50 (m. 1H, Ar-H), 10.67 (s, 1H, CH=N). ^13^C NMR: 28.0, 35.56, 36.46, 38.37 (adamantane-C), 49.41, 50.55 (piperazine-C), 68.82 (CH_2_), 110.76, 112.19, 116.31, 119.88, 129.10, 133.27, 151.38, 152.24 (Ar-C), 155.64, 161.06 (triazole C-5 & CH=N), 163.22 (C=S). MS, *m/z* (Rel. Int.): 550 (M^+^ +2, 36), 549 (M^+^ +1, 100).

*5-(1-Adamantyl)-4-(2,6-difluorobenzylideneamino)-2-[4-(4-fluorophenyl)-1-piperazinylmethyl]-1,2,4-triazoline-3-thione* **7e**: ^1^H-NMR (CDCl_3_): δ 1.80 (s, 6H, adamantane-H), 2.10 (s, 3H, adamantane-H), 2.19 (s, 6H, adamantane-H), 3.04 (s, 4H, piperazine-H), 3.14 (s, 4H, piperazine-H), 5.23 (s, 2H, CH_2_), 6.89–6.99 (m, 4H, Ar-H), 7.03 (t. 2H, Ar-H, *J* = 8.5 Hz), 7.48–7.49 (m, 1H, Ar-H), 10.66 (s, 1H, CH=N). ^13^C NMR: 28.0, 35.57, 36.46, 38.38 (adamantane-C), 50.40, 50.54 (piperazine-C), 68.78 (CH_2_), 110.85, 112.39, 115.41, 118.10, 133.28, 148.04, 152.24, 158.24 (Ar-C), 155.65 (CH=N), 161.06 (triazole C-5), 163.23 (C=S). MS, *m/z* (Rel. Int.): 568 (M^+^ +2, 33), 567 (M^+^ +1, 100).

*5-(1-Adamantyl)-4-(2,6-difluorobenzylideneamino)-2-[4-(3-trifluoromethylphenyl)-1-piperazinylmethyl]-1,2,4-triazoline-3-thione* **7f**: ^1^H-NMR (CDCl_3_): δ 1.80 (s, 6H, adamantane-H), 2.10 (s, 3H, adamantane-H), 2.19 (s, 6H, adamantane-H), 3.03-3.04 (m, 4H, piperazine-H), 3.21-3.27 (m, 4H, piperazine-H), 5.24 (s, 2H, CH_2_), 7.04–7.13 (m, 5H, Ar-H), 7.34 (t. 1H, Ar-H, *J* = 8.0 Hz), 7.46–7.50 (m, 1H, Ar-H), 10.67 (s, 1H, CH=N). ^13^C NMR: 27.99, 35.57, 36.44, 38.38 (adamantane-C), 48.87, 50.36 (piperazine-C), 68.73 (CH_2_), 118.86 (CF_3_), 110.83, 112.36, 115.09, 123.22, 125.39, 129.53, 131.58, 133.31, 151.44, 152.27 (Ar-C), 155.71, 161.01 (triazole C-5 & CH=N), 163.25 (C=S). MS, *m/z* (Rel. Int.): 618 (M^+^ +2, 35), 617 (M^+^ +1, 100).

*5-(1-Adamantyl)-4-(2,6-difluorobenzylideneamino)-2-[4-(2-methoxylphenyl)-1-piperazinylmethyl]-1,2,4-triazoline-3-thione* **7g**: ^1^H-NMR (CDCl_3_): δ 1.80 (s, 6H, adamantane-H), 2.10 (s, 3H, adamantane-H), 2.20 (s, 6H, adamantane-H), 3.10 (s, 8H, piperazine-H), 3.87 (s, 3H, OCH_3_), 5.25 (s, 2H, CH_2_), 6.86 (d, 1H, Ar-H, *J* = 7.5 Hz), 6.92–6.98 (m, 2H, Ar-H), 7.0–7.07 (m, 3H, Ar-H), 7.47–7.49 (m, 1H, Ar-H), 10.63 (s, 1H, CH=N). ^13^C NMR: 28.02, 35.55, 36.48, 38.34 (adamantane-C), 50.72, 50.83 (piperazine-C), 55.27 (OCH_3_), 69.04 (CH_2_), 110.03, 112.18, 112.38, 118.28, 120.94, 123.02, 133.23, 141.34, 152.26, 152.33 (Ar-C), 155.55, 161.02 (triazole C-5 & CH=N), 163.21 (C=S). MS, *m/z* (Rel. Int.): 580 (M^+^ +2, 33), 579 (M^+^ +1, 92).

*5-(1-Adamantyl)-4-(2,6-difluorobenzylideneamino)-2-(4-benzyl-1-piperazinylmethyl)-1,2,4-triazoline-3-thione* **7h**: ^1^H-NMR (CDCl_3_): δ 1.80 (s, 6H, adamantane-H), 2.09 (s, 3H, adamantane-H), 2.18 (s, 6H, adamantane-H), 2.41–2.52 (m, 4H, piperazine-H), 2.95 (s, 4H, piperazine-H), 3.52 (s, 2H, PhC***H***_2_), 5.17 (s, 2H, CH_2_), 7.03 (t, 2H, Ar-H, *J* = 8.5 Hz), 7.25–7.32 (m, 5H, Ar-H), 7.45–7.49 (m, 1H, Ar-H), 10.65 (s, 1H, CH=N). ^13^C NMR: 28.0, 35.52, 36.47, 38.36 (adamantane-C), 50.51, 53.12 (piperazine-C), 63.18 (Ph***C***H_2_), 68.96 (CH_2_), 110.89, 112.17, 127.04, 128.18, 129.29, 133.11, 137.96, 152.11 (Ar-C), 155.47, 161.05 (triazole C-5 & CH=N), 163.19 (C=S). MS, *m/z* (Rel. Int.): 564 (M^+^ +2, 34), 563 (M^+^ +1, 100).

*5-(1-Adamantyl)-4-(2,6-dichlorobenzylideneamino)-2-(4-methyl-1-piperazinylmethyl)-1,2,4-triazoline-3-thione* **7i**: ^1^H-NMR (CDCl_3_): δ 1.76 (s, 6H, adamantane-H), 2.07 (s, 3H, adamantane-H), 2.16 (s, 6H, adamantane-H), 2.33 (s, 3H, CH_3_), 2.50–2.54 (m, 4H, piperazine-H), 2.92–2.96 (m, 4H, piperazine-H), 5.17 (s, 2H, CH_2_), 7.22-7.48 (m, 3H, Ar-H), 10.80 (s, 1H, CH=N). ^13^C-NMR: 27.91, 35.54, 36.43, 38.44 (adamantane-C), 43.85 (CH_3_), 54.36, 56.42 (piperazine-C), 68.71 (CH_2_), 128.62, 129.43, 131.75, 136.18 (Ar-C), 155.42, 158.18 (triazole C-5 & CH=N), 163.44 (C=S). MS, *m/z* (Rel. Int.): 522 (M^+^ +4, 6), 520 (M^+^ +2, 55), 518 (M^+^, 100).

*5-(1-Adamantyl)-4-(2,6-dichlorobenzylideneamino)-2-(4-ethyl-1-piperazinylmethyl)-1,2,4-triazoline-3-thione* **7j**: ^1^H-NMR (CDCl_3_): δ 1.08 (t, 3H, CH_3_, *J* = 7.0 Hz), 1.75 (s, 6H, adamantane-H), 2.06 (s, 3H, adamantane-H), 2.16–2.17 (m, 6H, adamantane-H), 2.42 (q, 2H, C***H***_2_CH_3_, *J* = 7.0 Hz), 2.52–2.53 (m, 4H, piperazine-H), 2.95 (s, 4H, piperazine-H), 5.19 (s, 2H, CH_2_), 7.32-7.37 (m, 1H, Ar-H), 7.42–7.47 (m. 2H, Ar-H), 10.74 (s, 1H, CH=N). ^13^C-NMR: 11.87 (CH_3_), 27.96, 35.55, 36.47, 38.51 (adamantane-C), 50.41 (***C***H_2_CH_3_), 52.31, 52.76 (piperazine-C), 68.93 (CH_2_), 129.32, 129.40, 131.53, 136.10 (Ar-C), 155.41, 157.82 (triazole C-5 & CH=N), 163.42 (C=S). MS, *m/z* (Rel. Int.): 536 (M^+^ +4, 5), 534 (M^+^ +2, 43), 532 (M^+^, 100).

*5-(1-Adamantyl)-4-(2,6-dichlorobenzylideneamino)-2-(4-ethoxycarbonyl-1-piperazinylmethyl)-1,2,4-triazoline-3-thione* **7k**: ^1^H-NMR (CDCl_3_): δ 1.25 (t, 3H, CH_3_, *J* = 7.0 Hz), 1.76 (s, 6H, adamantane-H), 2.07 (s, 3H, adamantane-H), 2.16 (s, 6H, adamantane-H), 2.83 (s, 4H, piperazine-H), 3.51 (s, 4H, piperazine-H), 4.11 (q, 2H, C***H***_2_CH_3_, *J* = 7.0 Hz), 5.17 (s, 2H, CH_2_), 7.30–7.36 (m, 1H, Ar-H), 7.41–7.46 (m. 2H, Ar-H), 10.79 (s, 1H, CH=N). ^13^C-NMR: 14.64 (CH_3_), 27.94, 35.61, 36.44, 38.52 (adamantane-C), 50.30, 52.50 (piperazine-C), 61.33 (***C***H_2_CH_3_), 69.15 (CH_2_), 128.94, 129.37, 131.61, 136.09 (Ar-C), 155.30, 155.46, 157.76 (C=O, triazole C-5 & CH=N), 163.46 (C=S). MS, *m/z* (Rel. Int.): 599 (M^+^ +Na, 100), 577 (M^+^ +1, 7).

*5-(1-Adamantyl)-4-(2,6-dichlorobenzylideneamino)-2-(4-phenyl-1-piperazinylmethyl)-1,2,4-triazoline-3-thione* **7l**: ^1^H-NMR (CDCl_3_): δ 1.77 (s, 6H, adamantane-H), 2.08 (s, 3H, adamantane-H), 2.18 (s, 6H, adamantane-H), 3.06 (s, 4H, piperazine-H), 3.23 (s, 4H, piperazine-H), 5.25 (s, 2H, CH_2_), 6.87 (t, 1H, Ar-H, *J* = 7.5 Hz), 6.95 (d, 2H, Ar-H, *J* = 8.0 Hz), 7.27–7.37 (m, 4H, Ar-H), 7.46–7.47 (m, 1H, Ar-H), 10.80 (s, 1H, CH=N). ^13^C-NMR: 27.96, 35.61, 36.46, 38.54 (adamantane-C), 49.45, 50.60 (piperazine-C), 69.0 (CH_2_), 116.35, 119.92, 129.06, 129.11, 129.37, 131.60, 136.12, 151.39 (Ar-C), 155.61, 156.09, 157.83 (triazole C-5 & CH=N), 163.48 (C=S). MS, *m/z* (Rel. Int.): 585 (M^+^ +4, 16), 583 (M^+^ +2, 77), 581 (M^+^, 100).

*5-(1-Adamantyl)-4-(2,6-dichlorobenzylideneamino)-2-[4-(4-fluorophenyl)-1-piperazinylmethyl]-1,2,4-triazoline-3-thione* **7m**: ^1^H-NMR (CDCl_3_): δ 1.76 (s, 6H, adamantane-H), 2.08 (s, 3H, adamantane-H), 2.18 (s, 6H, adamantane-H), 3.05–3.06 (m, 4H, piperazine-H), 3.15 (s, 4H, piperazine-H), 5.24 (s, 2H, CH_2_), 6.88–6.91 (m, 2H, Ar-H), 6.96–6.99 (m, 2H, Ar-H), 7.33–7.36 (m, 1H, Ar-H), 7.45 (d, 2H, Ar-H, *J* = 7.0 Hz), 10.79 (s, 1H, CH=N). ^13^C-NMR: 27.96, 35.61, 36.46, 38.55 (adamantane-C), 50.44, 50.59 (piperazine-C), 68.95 (CH_2_), 115.43, 115.60, 118.08, 118.14, 129.37, 131.60, 136.11, 148.06 (Ar-C), 155.62, 157.83 (triazole C-5 & CH=N), 163.49 (C=S). MS, *m/z* (Rel. Int.): 602 (M^+^ +4, 23), 601 (M^+^ +3, 78), 600 (M^+^ +2, 32), 599 (M^+^ +1, 100).

*5-(1-Adamantyl)-4-(2,6-dichlorobenzylideneamino)-2-[4-(3-trifluoromethylphenyl)-1-piperazinylmethyl]-1,2,4-triazoline-3-thione* **7n**: ^1^H-NMR (CDCl_3_): δ 1.77 (s, 6H, adamantane-H), 2.08 (s, 3H, adamantane-H), 2.18 (s, 6H, adamantane-H), 3.05–3.06 (m, 4H, piperazine-H), 3.27–3.28 (m, 4H, piperazine-H), 5.24 (s, 2H, CH_2_), 7.07 (t, 2H, Ar-H, *J* = 8.5 Hz), 7.14 (s, 1H, Ar-H), 7.34–7.37 (m, 2H, Ar-H), 7.46 (d, 2H, Ar-H, *J* = 8.0 Hz), 10.80 (s, 1H, CH=N). ^13^C-NMR: 27.94, 35.62, 36.44, 38.55 (adamantane-C), 48.89, 50.40 (piperazine-C), 68.91 (CH_2_), 118.89 (CF_3_), 112.43, 115.98, 123.22, 125.39, 129.02, 129.39, 129.55, 131.78, 136.12, 151.44 (Ar-C), 155.68, 157.86 (triazole C-5 & CH=N), 163.50 (C=S). MS, *m/z* (Rel. Int.): 652 (M^+^ +4, 16), 651 (M^+^ +3, 76), 650 (M^+^ +2, 32), 649 (M^+^ +1, 100).

*5-(1-Adamantyl)-4-(2,6-dichlorobenzylideneamino)-2-[4-(2-methoxyphenyl)-1-piperazinylmethyl]-1,2,4-triazoline-3-thione* **7o**: ^1^H-NMR (CDCl_3_): δ 1.77 (s, 6H, adamantane-H), 2.08 (s, 3H, adamantane-H), 2.19 (s, 6H, adamantane-H), 3.11 (s, 8H, piperazine-H), 3.87 (s, 3H, OCH_3_), 5.26 (s, 2H, CH_2_), 6.86 (d, 1H, Ar-H, *J* = 7.5 Hz), 6.93–7.04 (m, 3H, Ar-H), 7.33–7.36 (m, 1H, Ar-H) 7.45 (d, 2H, Ar-H, *J* = 8.0 Hz), 10.76 (s, 1H, CH=N). ^13^C-NMR: 27.98, 35.59, 36.49, 38.51 (adamantane-C), 50.76, 50.84 (piperazine-C), 55.28 (OCH_3_), 69.22 (CH_2_), 110.98, 118.29, 120.93, 123.04, 129.12, 129.34, 131.55, 136.12, 141.32, 152.27 (Ar-C), 155.50 (triazole C-5), 157.89 (CH=N), 163.49 (C=S). MS, *m/z* (Rel. Int.): 614 (M^+^ +4, 9), 613 (M^+^ +3, 35), 612 (M^+^ +2, 14), 611 (M^+^ +1, 46).

*5-(1-Adamantyl)-4-(2,6-dichlorobenzylideneamino)-2-(4-benzyl-1-piperazinylmethyl)-1,2,4-triazoline-3-thione* **7p**: ^1^H-NMR (CDCl_3_): δ 1.76 (s, 6H, adamantane-H), 2.07 (s, 3H, adamantane-H), 2.17 (s, 6H, adamantane-H), 2.51 (s, 4H, piperazine-H), 2.92 (s, 4H, piperazine-H), 3.52 (s, 2H, PhC***H***_2_), 5.17 (s, 2H, CH_2_), 7.26–7.36 (m, 6H, Ar-H), 7.45 (d, 2H, Ar-H , *J* = 8.0 Hz), 10.78 (s, 1H, CH=N). ^13^C-NMR: 27.96, 35.56, 36.47, 38.53 (adamantane-C), 50.53, 53.14 (piperazine-C), 63.24 (Ph***C***H_2_), 69.11 (CH_2_), 127.07, 128.20, 129.12, 129.33, 129.42, 131.54, 136.10, 137.90 (Ar-C), 155.45, 157.68 (triazole C-5 & CH=N), 163.45 (C=S). MS, *m/z* (Rel. Int.): 598 (M^+^ +4, 13), 597 (M^+^ +3, 76), 596 (M^+^ +2, 35), 595 (M^+^ +1, 100).

### 5-(1-Adamantyl)-4-arylideneamino-2-(4-ethoxycarbonyl-1-piperidylmethyl)-1,2,4-triazoline-3-thiones ***8a–n***

A mixture of the 5-(1-adamantyl)-4-arylideneamino-3-mercapto-1,2,4-triazole **6** (1.0 mmol), ethyl 4-piperidinecarboxylate (0.16 g, 1.0 mmol) and 37% formaldehyde solution (1 mL), in ethanol (8 mL), was heated under reflux for 20 min when a clear solution was obtained. Stirring was continued for 12 h at room temperature and the mixture was allowed to stand overnight. Cold water (5 mL) was added and the reaction mixture was stirred for 20 min. The precipitated crude products were filtered, washed with water, dried, and crystallized (Table 3).

*5-(1-Adamantyl)-4-benzylideneamino-2-(4-ethoxycarbonyl-1-piperidylmethyl)-1,2,4-triazoline-3-thione*** 8a**: ^1^H-NMR (CDCl_3_): δ 1.24 (t, 3H, CH_3_, *J* = 7.0 Hz), 1.72–1.83 (m, 8H, 6 adamantane-H & 2 piperidine-H), 1.92–1.94 (m, 2H, piperidine-H), 2.10 (s, 3H, adamantane-H), 2.17 (s, 6H, adamantane-H), 2.20–2.25 (m, 1H, piperidine-4 H), 2.46–2.50 (m, 2H, piperidine-H), 3.20–3.22 (m, 2H, piperidine-H), 4.12 (q, 2H, C***H***_2_CH_3_, *J* = 7.0 Hz), 5.15 (s, 2H, CH_2_), 7.51–7.58 (m, 3H, Ar-H), 7.91 (d, 2H, Ar-H, *J* = 7.0 Hz), 10.08 (s, 1H, CH=N). ^13^C-NMR: 14.20 (CH_3_), 27.93, 35.44, 36.58, 38.77 (adamantane-C), 28.35 (piperidine C-3), 40.42 (piperidine C-4), 50.42 (piperidine C-2), 60.25 (***C***H_2_CH_3_), 69.63 (CH_2_), 128.72, 129.02, 132.28, 132.84 (Ar-C), 155.28, 162.26 (triazole C-5 & CH=N), 163.21 (C=S), 174.99 (C=O). MS, *m/z* (Rel. Int.): 509 (M^+^ +2, 21), 508 (M^+^ +1, 72).

*5-(1-Adamantyl)-4-(2-fluorobenzylideneamino)-2-(4-ethoxycarbonyl-1-piperidylmethyl)-1,2,4-triazoline-3-thione*** 8b**: ^1^H-NMR (CDCl_3_): δ 1.24 (t, 3H, C***H***_3_CH_2_, *J* = 7.0 Hz), 1.72–1.82 (m, 8H, 6 adamantane-H & 2 piperidine-H), 1.92–1.94 (m, 2H, piperidine-H), 2.10 (s, 3H, adamantane-H), 2.16 (s, 6H, adamantane-H), 2.20–2.25 (m, 1H, piperidine-4 H), 2.50–2.54 (m, 2H, piperidine-H), 3.19–3.22 (m, 2H, piperidine-H), 4.12 (q, 2H, C***H***_2_CH_3_, *J* = 7.0 Hz), 5.15 (s, 2H, CH_2_), 7.18 (t, 1H, Ar-H, *J* = 8.0 Hz), 7.30 (d, 1H, Ar-H, *J* = 7.5 Hz), 7.53–7.56 (m, 1H, Ar-H), 8.08-8.11 (m, 1H, Ar-H), 10.42 (s, 1H, CH=N). ^13^C-NMR: 14.20 (CH_3_), 27.93, 35.46, 36.56, 38.76 (adamantane-C), 28.35 (piperidine C-3), 40.77 (piperidine C-4), 50.42 (piperidine C-2), 60.25 (***C***H_2_CH_3_), 69.64 (CH_2_), 116.31, 120.89, 124.66, 127.57, 133.90, 155.82 (Ar-C), 155.28, 161.54 (triazole C-5 & CH=N), 163.31 (C=S), 174.99 (C=O). MS, *m/z* (Rel. Int.): (M^+^ +2, 33), 526 (M^+^ +1, 100).

*5-(1-Adamantyl)-4-(2-chlorobenzylideneamino)-2-(4-ethoxycarbonyl-1-piperidylmethyl)-1,2,4-triazoline-3-thione*** 8c**: ^1^H-NMR (CDCl_3_): δ 1.24 (t, 3H, C***H***_3_CH_2_, *J* = 7.0 Hz), 1.72–1.79 (m, 8H, 6 adamantane-H & 2 piperidine-H), 1.92–1.94 (m, 2H, piperidine-H), 2.11 (s, 3H, adamantane-H), 2.17 (s, 6H, adamantane-H), 2.21–2.25 (m, 1H, piperidine-4 H), 2.51-2.55 (m, 2H, piperidine-H), 3.20–3.22 (m, 2H, piperidine-H), 4.12 (q, 2H, C***H***_2_CH_3_, *J* = 7.0 Hz), 5.15 (s, 2H, CH_2_), 7.40 (t, 1H, Ar-H, *J* = 7.5 Hz), 7.46–7.51 (m, 2H, Ar-H), 8.19 (d, 1H, Ar-H, *J* = 7.5 Hz), 10.69 (s, 1H, CH=N). ^13^C-NMR: 14.20 (CH_3_), 27.93, 35.49, 36.58, 38.83 (adamantane-C), 28.35 (piperidine C-3), 40.78 (piperidine C-4), 50.43 (piperidine C-2), 60.25 (***C***H_2_CH_3_), 69.65 (CH_2_), 127.20, 127.60, 130.34, 130.85, 132.97, 136.68 (Ar-C), 155.28, 158.27 (triazole C-5 & CH=N), 163.37 (C=S), 174.99 (C=O). MS, *m/z* (Rel. Int.): 564 (M^+^ +Na, 100), 544 (23), 542 (54).

*5-(1-Adamantyl)-4-(4-methylbenzylideneamino)-2-(4-ethoxycarbonyl-1-piperidylmethyl)-1,2,4-triazoline-3-thione*** 8d**: ^1^H-NMR (CDCl_3_): δ 1.24 (t, 3H, C***H***_3_CH_2_, *J* = 7.0 Hz), 1.72–1.78 (m, 8H, 6 adamantane-H & 2 piperidine-H), 1.91–1.94 (m, 2H, piperidine-H), 2.09 (s, 3H, adamantane-H), 2.16 (s, 6H, adamantane-H), 2.20–2.25 (m, 1H, piperidine-4 H), 2.45 (s, 3H, PhC***H***_3_), 2.50–2.54 (m, 2H, piperidine-H), 3.19–3.22 (m, 2H, piperidine-H), 4.12 (q, 2H, C***H***_2_CH_3_, *J* = 7.0 Hz), 5.15 (s, 2H, CH_2_), 7.31 (d, 2H, Ar-H, *J* = 8.0 Hz), 7.80 (d, 2H, Ar-H, *J* = 8.0 Hz), 9.94 (s, 1H, CH=N). ^13^C-NMR: 14.20 (CH_3_), 21.71 (Ph***C***H_3_), 27.93, 35.40, 36.58, 38.75 (adamantane-C), 28.35 (piperidine C-3), 40.78 (piperidine C-4), 50.41 (piperidine C-2), 60.24 (***C***H_2_CH_3_), 69.62 (CH_2_), 128.75, 129.76, 130.10, 143.05 (Ar-C), 155.24, 162.69 (triazole C-5 & CH=N), 163.21 (C=S), 174.99 (C=O). MS, *m/z* (Rel. Int.): 544 (M^+^ +Na, 100), 523 (M^+^ +2, 43), 522 (M^+^ +1, 76).

*5-(1-Adamantyl)-4-(2-hydroxybenzylideneamino)-2-(4-ethoxycarbonyl-1-piperidylmethyl)-1,2,4-triazoline-3-thione*** 8e**: ^1^H-NMR (CDCl_3_): δ 1.25 (t, 3H, C***H***_3_CH_2_, *J* = 7.0 Hz), 1.73–1.79 (m, 8H, 6 adamantane-H & 2 piperidine-H), 1.92–1.95 (m, 2H, piperidine-H), 2.10 (s, 9H, adamantane-H), 2.22–2.24 (m, 1H, piperidine-4 H), 2.51–2.55 (m, 2H, piperidine-H), 3.19–3.21 (m, 2H, piperidine-H), 4.12 (q, 2H, C***H***_2_CH_3_, *J* = 7.0 Hz), 5.15 (s, 2H, CH_2_), 7.03–7.06 (m, 1H, Ar-H), 7.08 (d, 1H, Ar-H, *J* = 8.5 Hz), 7.44–7.51 (m, 2H, Ar-H), 9.69 (s, 1H, CH=N), 10.45 (s, 1H, OH). ^13^C-NMR: 14.20 (CH_3_), 27.80, 35.35, 36.29, 38.97 (adamantane-C), 28.32 (piperidine C-3), 40.73 (piperidine C-4), 50.42 (piperidine C-2), 60.28 (***C***H_2_CH_3_), 70.22 (CH_2_), 116.09, 117.56, 120.09, 133.60, 134.69, 169.08 (Ar-C), 154.28, 160.08 (triazole C-5 & CH=N), 164.01 (C=S), 174.94 (C=O). MS, *m/z* (Rel. Int.): 546 (M^+^ +Na, 100), 525 (M^+^ +2, 26), 524 (M^+^ +1, 84).

*5-(1-Adamantyl)-4-(4-hydroxybenzylideneamino)-2-(4-ethoxycarbonyl-1-piperidylmethyl)-1,2,4-triazoline-3-thione*** 8f**: ^1^H-NMR (CDCl_3_): δ 1.25 (t, 3H, C***H***_3_CH_2_, *J* = 7.0 Hz), 1.75–1.78 (m, 8H, 6 adamantane-H & 2 piperidine-H), 1.94–1.96 (m, 2H, piperidine-H), 2.07 (s, 3H, adamantane-H), 2.12 (s, 6H, adamantane-H), 2.24–2.28 (m, 1H, piperidine-4 H), 2.52–2.56 (m, 2H, piperidine-H), 3.21–3.23 (m, 2H, piperidine-H), 4.13 (q, 2H, C***H***_2_CH_3_, *J* = 7.0 Hz), 5.14 (s, 2H, CH_2_), 6.92 (d, 2H, Ar-H, *J* = 8.5 Hz), 7.72 (d, 2H, Ar-H, *J* = 8.5 Hz), 9.55 (s, 1H, CH=N). ^13^C-NMR: 14.17 (CH_3_), 27.88, 35.37, 36.52, 38.66 (adamantane-C), 28.15 (piperidine C-3), 40.72 (piperidine C-4), 50.43 (piperidine C-2), 58.53 (***C***H_2_CH_3_), 69.64 (CH_2_), 116.24, 124.64, 130.91, 163.01 (Ar-C), 155.38, 160.42 (triazole C-5 & CH=N), 164.17 (C=S), 175.32 (C=O). MS, *m/z* (Rel. Int.): 525 (M^+^ +2, 33), 524 (M^+^ +1, 100).

*5-(1-Adamantyl)-4-(4-methoxybenzylideneamino)-2-(4-ethoxycarbonyl-1-piperidylmethyl)-1,2,4-triazoline-3-thione*** 8g**: ^1^H-NMR (CDCl_3_): δ 1.24 (t, 3H, C***H***_3_CH_2_, *J* = 7.0 Hz), 1.72–1.79 (m, 8H, 6 adamantane-H & 2 piperidine-H), 1.91–1.93 (m, 2H, piperidine-H), 2.09 (s, 3H, adamantane-H), 2.16 (s, 6H, adamantane-H), 2.20–2.24 (m, 1H, piperidine-4 H), 2.50-2.53 (m, 2H, piperidine-H), 3.19–3.21 (m, 2H, piperidine-H), 3.90 (s, 3H, OCH_3_), 4.12 (q, 2H, C***H***_2_CH_3_, *J* = 7.0 Hz), 5.15 (s, 2H, CH_2_), 7.02 (d, 2H, Ar-H, *J* = 8.5 Hz), 7.86 (d, 2H, Ar-H , *J* = 8.5 Hz), 9.81 (s, 1H, CH=N). ^13^C-NMR: 14.20 (CH_3_), 27.93, 35.37, 36.58, 38.74 (adamantane-C), 28.35 (piperidine C-3), 40.79 (piperidine C-4), 55.48 (piperidine C-2), 60.23 (***C***H_2_CH_3_), 69.64 (CH_2_), 114.54, 125.35, 130.59, 162.76 (Ar-C), 155.18, 162.69 (triazole C-5 & CH=N), 163.21 (C=S), 174.99 (C=O). MS, *m/z* (Rel. Int.): 560 (M^+^ +Na, 100), 539 (M^+^ +2, 38), 538 (M^+^ +1, 86).

*5-(1-Adamantyl)-4-(2,6-difluorobenzylideneamino)-2-(4-ethoxycarbonyl-1-piperidylmethyl)-1,2,4-triazoline-3-thione*** 8h**: ^1^H-NMR (CDCl_3_): δ 1.24 (t, 3H, C***H***_3_CH_2_, *J* = 7.0 Hz), 1.71–1.80 (m, 8H, 6 adamantane-H & 2 piperidine-H), 1.92–1.94 (m, 2H, piperidine-H), 2.09 (s, 3H, adamantane-H), 2.18 (s, 6H, adamantane-H), 2.21–2.25 (m, 1H, piperidine-4 H), 2.50–2.54 (m, 2H, piperidine-H), 3.19–3.21 (m, 2H, piperidine-H), 4.12 (q, 2H, C***H***_2_CH_3_, *J* = 7.0 Hz), 5.14 (s, 2H, CH_2_), 7.03 (t, 2H, Ar-H, *J* = 8.5 Hz), 7.45–7.49 (m, 1H, Ar-H), 10.70 (s, 1H, CH=N). ^13^C-NMR: 14.19 (CH_3_), 28.0, 35.53, 36.46, 38.41 (adamantane-C), 28.35 (piperidine C-3), 40.77 (piperidine C-4), 50.42 (piperidine C-2), 60.25 (***C***H_2_CH_3_), 69.43 (CH_2_), 110.89, 112.37, 133.21, 161.04 (Ar-C), 155.53, 160.99 (triazole C-5 & CH=N), 163.21 (C=S), 174.98 (C=O). MS, *m/z* (Rel. Int.): 566 (M^+^ +Na, 100), 545 (M^+^ +2, 18), 544 (M^+^ +1, 61).

*5-(1-Adamantyl)-4-(2-chloro-6-fluorobenzylideneamino)-2-(4-ethoxycarbonyl-1-piperidylmethyl)-1,2,4-triazoline-3-thione*** 8i**: ^1^H-NMR (CDCl_3_): δ 1.24 (t, 3H, C***H***_3_CH_2_, *J* = 7.0 Hz), 1.71–1.79 (m, 8H, 6 adamantane-H & 2 piperidine-H), 1.92–1.94 (m, 2H, piperidine-H), 2.09 (s, 3H, adamantane-H), 2.18 (s, 6H, adamantane-H), 2.21–2.25 (m, 1H, piperidine-4 H), 2.51–2.55 (m, 2H, piperidine-H), 3.20–3.22 (m, 2H, piperidine-H), 4.12 (q, 2H, C***H***_2_CH_3_, *J* = 7.0 Hz), 5.15 (s, 2H, CH_2_), 7.14–7.17 (m, 2H, Ar-H), 7.33 (d, 1H, Ar-H, *J* = 7.5 Hz), 10.87 (s, 1H, CH=N). ^13^C-NMR: 14.19 (CH_3_), 28.0, 35.55, 36.46, 38.40 (adamantane-C), 28.36 (piperidine C-3), 40.77 (piperidine C-4), 50.43 (piperidine C-2), 60.24 (***C***H_2_CH_3_), 69.46 (CH_2_), 115.35, 119.81, 126.25, 132.52, 137.10, 162.85 (Ar-C), 155.11 (CH=N), 160.76 (triazole C-5), 163.05 (C=S), 174.99 (C=O). MS, *m/z* (Rel. Int.): 582 (M^+^ +Na, 100), 562 (29), 561 (M^+^ +2, 21), 560 (M^+^ +1, 63).

*5-(1-Adamantyl)-4-(2,6-dichlorobenzylideneamino)-2-(4-ethoxycarbonyl-1-piperidylmethyl)-1,2,4-triazoline-3-thione*** 8j**: ^1^H-NMR (CDCl_3_): δ 1.24 (t, 3H, C***H***_3_CH_2_, *J* = 7.0 Hz), 1.72–1.81 (m, 8H, 6 adamantane-H & 2 piperidine-H), 1.92–1.95 (m, 2H, piperidine-H), 2.07 (s, 3H, adamantane-H), 2.17 (s, 6H, adamantane-H), 2.21–2.26 (m, 1H, piperidine-4 H), 2.53–2.57 (m, 2H, piperidine-H), 3.20–3.22 (m, 2H, piperidine-H), 4.12 (q, 2H, C***H***_2_CH_3_, *J* = 7.0 Hz), 5.15 (s, 2H, CH_2_), 7.33–7.36 (m, 1H, Ar-H), 7.45 (d, 2H, Ar-H, *J* = 8.0 Hz), 10.84 (s, 1H, CH=N). ^13^C-NMR: 14.20 (CH_3_), 27.96, 35.59, 36.46, 38.58 (adamantane-C), 28.36 (piperidine C-3), 40.77 (piperidine C-4), 50.44 (piperidine C-2), 60.24 (***C***H_2_CH_3_), 69.62 (CH_2_), 128.77, 129.36, 131.54, 136.08 (Ar-C), 155.52, 157.56 (triazole C-5 & CH=N), 163.22 (C=S), 174.99 (C=O). MS, *m/z* (Rel. Int.): 598 (M^+^ +Na, 100), 578 (52), 577 (M^+^ +2, 23), 576 (M^+^ +1, 73).

*5-(1-Adamantyl)-4-(2,4-dichlorobenzylideneamino)-2-(4-ethoxycarbonyl-1-piperidylmethyl)-1,2,4-triazoline-3-thione*** 8k**: ^1^H-NMR (CDCl_3_): δ 1.24 (t, 3H, C***H***_3_CH_2_, *J* = 7.0 Hz), 1.72–1.83 (m, 8H, 6 adamantane-H & 2 piperidine-H), 1.91–1.94 (m, 2H, piperidine-H), 2.11 (s, 3H, adamantane-H), 2.16 (s, 6H, adamantane-H), 2.20–2.25 (m, 1H, piperidine-4 H), 2.50–2.54 (m, 2H, piperidine-H), 3.19–3.21 (m, 2H, piperidine-H), 4.12 (q, 2H, C***H***_2_CH_3_, *J* = 7.0 Hz), 5.14 (s, 2H, CH_2_), 7.40 (d, 1H, Ar-H, *J* = 8.5 Hz), 7.53 (s, 1H, Ar-H), 8.11 (d, 1H, Ar-H, *J* = 8.5 Hz), 10.73 (s, 1H, CH=N). ^13^C-NMR: 14.20 (CH_3_), 27.91, 35.50, 36.57, 38.86 (adamantane-C), 28.34 (piperidine C-3), 40.76 (piperidine C-4), 50.42 (piperidine C-2), 60.26 (***C***H_2_CH_3_), 69.62 (CH_2_), 127.88, 128.28, 129.55, 130.16, 137.15, 138.58 (Ar-C), 155.23, 156.68 (triazole C-5 & CH=N), 163.33 (C=S), 174.96 (C=O). MS, *m/z* (Rel. Int.): 598 (M^+^ +Na, 100), 578 (56), 577 (M^+^ +2, 24), 576 (M^+^ +1, 78).

*5-(1-Adamantyl)-4-(3,4-dichlorobenzylideneamino)-2-(4-ethoxycarbonyl-1-piperidylmethyl)-1,2,4-triazoline-3-thione*** 8l**: ^1^H-NMR (CDCl_3_): δ 1.24 (t, 3H, C***H***_3_CH_2_, *J* = 7.0 Hz), 1.71–1.84 (m, 8H, 6 adamantane-H & 2 piperidine-H), 1.91–1.93 (m, 2H, piperidine-H), 2.12 (s, 3H, adamantane-H), 2.15 (s, 6H, adamantane-H), 2.20–2.24 (m, 1H, piperidine-4 H), 2.49–2.53 (m, 2H, piperidine-H), 3.18–3.20 (m, 2H, piperidine-H), 4.11 (q, 2H, C***H***_2_CH_3_, *J* = 7.0 Hz), 5.13 (s, 2H, CH_2_), 7.59 (d, 1H, Ar-H, *J* = 8.0 Hz), 7.72 (d, 1H, Ar-H, *J* = 8.0 Hz), 7.98 (s, 1H, Ar-H), 10.29 (s, 1H, CH=N). ^13^C-NMR: 14.20 (CH_3_), 27.90, 35.50, 36.57, 38.86 (adamantane-C), 28.33 (piperidine C-3), 40.74 (piperidine C-4), 50.41 (piperidine C-2), 60.27 (***C***H_2_CH_3_), 69.63 (CH_2_), 127.44, 130.03, 131.16, 133.01, 133.67, 136.40 (Ar-C), 155.25, 158.17 (triazole C-5 & CH=N), 163.20 (C=S), 174.94 (C=O). MS, *m/z* (Rel. Int.): 598 (M^+^ +Na, 100), 577 (M^+^ +2, 20), 576 (M^+^ +1, 67).

*5-(1-Adamantyl)-4-(3,4-dimethoxybenzylideneamino)-2-(4-ethoxycarbonyl-1-piperidylmethyl)-1,2,4-triazoline-3-thione*** 8m**: ^1^H-NMR (CDCl_3_): δ 1.24 (t, 3H, C***H***_3_CH_2_, *J* = 7.0 Hz), 1.76-1.82 (m, 8H, 6 adamantane-H & 2 piperidine-H), 1.91-1.93 (m, 2H, piperidine-H), 2.09 (s, 3H, adamantane-H), 2.17 (s, 6H, adamantane-H), 2.20-2.24 (m, 1H, piperidine-4 H), 2.49-2.54 (m, 2H, piperidine-H), 3.19-3.21 (m, 2H, piperidine-H), 3.96 (s, 3H, OCH_3_), 3.98 (s, 3H, OCH_3_), 4.11 (q, 2H, C***H***_2_CH_3_, *J* = 7.0 Hz), 5.15 (s, 2H, CH_2_), 6.79 (d, 1H, Ar-H, *J* = 8.0 Hz), 7.40 (d, 1H, Ar-H, *J* = 8.0 Hz), 7.54 (s, 1H, Ar-H), 9.85 (s, 1H, CH=N). ^13^C-NMR: 14.19 (CH_3_), 27.94, 35.39, 36.61, 38.80 (adamantane-C), 28.35 (piperidine C-3), 40.78 (piperidine C-4), 50.41 (piperidine C-2), 55.92 (OCH_3_), 56.08 (OCH_3_), 60.24 (***C***H_2_CH_3_), 69.63 (CH_2_), 109.19, 110.96, 124.42, 125.61, 149.60, 152.98 (Ar-C), 155.13, 162.48 (triazole C-5 & CH=N), 163.18 (C=S), 174.98 (C=O). MS, *m/z* (Rel. Int.): 590 (M^+^ +Na, 100), 569 (M^+^ +2, 26), 568 (M^+^ +1, 84).

*5-(1-Adamantyl)-4-(4,5-dimethoxy-2-nitrobenzylideneamino)-2-(4-ethoxycarbonyl-1-piperidylmethyl)-1,2,4-triazoline-3-thione*** 8n**: ^1^H-NMR (CDCl_3_): δ 1.24 (t, 3H, C***H***_3_CH_2_, *J* = 7.0 Hz), 1.73–1.80 (m, 8H, 6 adamantane-H & 2 piperidine-H), 1.92–1.94 (m, 2H, piperidine-H), 2.08 (s, 3H, adamantane-H), 2.15 (s, 6H, adamantane-H), 2.21–2.23 (m, 1H, piperidine-4 H), 2.51–2.55 (m, 2H, piperidine-H), 3.18–3.21 (m, 2H, piperidine-H), 4.04 (s, 3H, OCH_3_), 4.05 (s, 3H, OCH_3_), 4.11 (q, 2H, C***H***_2_CH_3_, *J* = 7.0 Hz), 5.14 (s, 2H, CH_2_), 7.65 (s, 1H, Ar-H), 7.63 (s, 1H, Ar-H), 10.54 (s, 1H, CH=N). ^13^C-NMR: 14.20 (CH_3_), 27.89, 35.42, 36.51, 38.81 (adamantane-C), 28.34 (piperidine C-3), 40.75 (piperidine C-4), 50.41 (piperidine C-2), 56.60 (OCH_3_), 56.68 (OCH_3_), 60.25 (***C***H_2_CH_3_), 69.85 (CH_2_), 107.82, 110.0, 122.50, 142.33, 151.45, 153.30 (Ar-C), 155.03, 160.57 (triazole C-5 & CH=N), 163.57 (C=S), 174.99 (C=O). MS, *m/z* (Rel. Int.): 635 (M^+^ +Na, 100), 614 (M^+^ +2, 23), 613 (M^+^ +1, 68).

*Determination of the antimicrobial activity by the agar disc-diffusion method.* Sterile filter paper discs (8 mm diameter) were moistened with the compound solution in dimethylsulphoxide of specific concentration (200 μg/disc), the antibacterial antibiotics Gentamicin and Ampicillin trihydrate (100 μg/disc) and the antifungal drug Clotrimazole (100 μg/disc) were carefully placed on the agar culture plates that had been previously inoculated separately with the microorganisms. The plates were incubated at 37 ºC, and the diameter of the growth inhibition zones were measured after 24 h in case of bacteria and 48 h in case of *Candida albicans*.

*Determination of minimal inhibitory concentration (MIC)*. Compounds **6h**, **6o**, **7n**, **8a**, **8e**, **8f** and **8m**, Gentamicin, Ampicillin trihydrate and Clotrimazole were dissolved in dimethylsulphoxide at concentration of 128 μg/mL. The twofold dilutions of the solution were prepared (128, 64, 32, …, 0.5 μg/mL). The microorganism suspensions at 106 CFU/mL (colony forming unit/ml) concentrations were inoculated to the corresponding wells. The plates were incubated at 36 ºC for 24 and 48 h for the bacteria and *Candida albicans*, respectively. The MIC values were determined as the lowest concentration that completely inhibited visible growth of the microorganism as detected by unaided eye.

*Determination of the anti-inflammatory activity*. Male Sprague-Dawley rats weighing 140–190 g were maintained at room temperature (20–23 ºC). The animals were randomly divided into 42 groups each of 5 animals. The animals were housed with food and water *ad libitum* and allowed to be accustomed to their environment for two days before testing. Each group was injected with the specific dose of the test compound (20 and 40 mg/kg), or Indomethacin (5 mg/kg) intraperitoneally as a uniform suspension in 1 ml of 0.5% (w/v) aqueous carboxymethyl cellulose solution, one hour before injection of 0.1 mL of carrageenan (1% solution in normal saline) into the plantar tissue of the right hind paw. The left hind paw was injected with 0.1 mL of normal saline solution. Four hours after carrageenan injection, the volume of paw oedema (mL) was determined using water plethysmometer. The percentage protection against inflammation was calculated as follows:

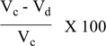



Where V_c_ is the mean percentage increase in paw volume in the absence of the test compound (control) and V_d_ is the mean percentage increase in paw volume after injection of the test compound. The values are expressed as the mean percentage reduction ± S.E.M. Statistical significance between the control and treated groups was performed using the Student “*t*” test.

## 4. Conclusions

In this study, new series of 5-(1-adamantyl)-4-arylideneamino-3-mercapto-1,2,4-triazoles **6a-v** , the *N*-Mannich bases 5-(1-adamantyl)-4-arylideneamino-2-(4-substituted-1-piperazinylmethyl)-1,2,4-triazoline-3-thiones **7a–p** and 5-(1-adamantyl)-4-arylideneamino-2-(4-ethoxycarbonyl-1-piperidyl-methyl)-1,2,4-triazoline-3-thiones **8a-n**, were synthesized and their antimicrobial and anti-inflammatory activity was determined. Several of the newly synthesized derivatives displayed promising antimicrobial and anti-inflammatory activities compared to known antibacterial, antifungal and anti-inflammatory drugs. Though, the mechanism of the biological activity needs further investigations, which are in progress.
